# The identification of differentially expressed genes in male and female gametophytes of simple thalloid liverwort *Pellia endiviifolia* sp. B using an RNA-seq approach

**DOI:** 10.1007/s00425-020-03424-z

**Published:** 2020-07-15

**Authors:** Izabela Sierocka, Sylwia Alaba, Artur Jarmolowski, Wojciech M. Karlowski, Zofia Szweykowska-Kulinska

**Affiliations:** 1 Department of Gene Expression, Institute of Molecular Biology and Biotechnology, Faculty of Biology, Adam Mickiewicz University in Poznan, Uniwersytetu Poznanskiego 6, 61-614 Poznan, Poland; 2 Department of Computational Biology, Institute of Molecular Biology and Biotechnology, Faculty of Biology, Adam Mickiewicz University in Poznan, Uniwersytetu Poznanskiego 6, 61-614 Poznan, Poland

**Keywords:** Antheridia, Archegonia, Bryophytes, Generative phase, Thalli, Vegetative phase

## Abstract

**Main conclusion:**

**This study shows differences in gene expression between male and female gametophytes of the simple thalloid liverwort with a distinction between the vegetative and reproductive phases of growth.**

**Abstract:**

*Pellia endiviifolia* is a simple thalloid liverwort that, together with hornworts and mosses, represents the oldest living land plants. The limited taxon sampling for genomic and functional studies hampers our understanding of processes governing evolution of these plants. RNA sequencing represents an attractive way to elucidate the molecular mechanisms of non-model species development. In the present study, RNA-seq was used to profile the differences in gene expression between *P. endiviifolia* male and female gametophytes, with a distinction between the vegetative and reproductive phases of growth. By comparison of the gene expression profiles from individuals producing sex organs with the remaining thalli types, we have determined a set of genes whose expression might be important for the development of *P. endiviifolia* reproductive organs. The selected differentially expressed genes (DEGs) were categorized into five main pathways: metabolism, genetic information processing, environmental information processing, cellular processes, and organismal systems. A comparison of the obtained data with the *Marchantia polymorpha* transcriptome resulted in the identification of genes exhibiting a similar expression pattern during the reproductive phase of growth between members of the two distinct liverwort classes. The common expression profile of  87 selected genes suggests a common mechanism governing sex organ development in both liverwort species. The obtained RNA-seq results were confirmed by RT-qPCR for the DEGs with the highest differences in expression level. Five *Pellia*-female-specific and two *Pellia*-male-specific DEGs showed enriched expression in archegonia and antheridia, respectively. The identified genes are promising candidates for functional studies of their involvement in liverwort sexual reproduction.

**Electronic supplementary material:**

The online version of this article (10.1007/s00425-020-03424-z) contains supplementary material, which is available to authorized users.

## Introduction

The colonization of the terrestrial environment by the first land plants represents an evolutionary milestone that changed the evolutionary course of life and the ecosystems on Earth. The transition from freshwater to land by plants probably occurred in a stepwise fashion, starting over 500 million years ago (Morris et al. 2018). The fossil records of cryptospores and the earliest plant fragments indicate plants with a bryophyte body plan organization (Wellman et al. 2003; Rubinstein et al. 2010; Brown et al. 2015) most likely resembling today’s liverworts. Bryophytes—hornworts, liverworts, and mosses—are considered to be the earliest diverging land plants and, as the remnants of early land colonization, provide a living laboratory for research on the morphological adaptations that enabled them to survive in a terrestrial environment (Supplementary Data S1: Fig. S1). Out of the 93 assembled and annotated genomes hosted in the Phytozome v12.1.6 database, there are 82 genomes of Viridiplantae species and only two bryophyte genomes, with *Physcomitrella patens* and *Marchantia polymorpha* being the leading model organisms for mosses and liverworts, respectively (Rensing et al. 2008; Bowman et al. 2017; Lang et al. 2018). The application of various molecular genetics tools in both of these species has been successful in revealing the evolutionary course through several developmental mechanisms that regulate different morphological traits (Menand et al. 2007; Yasamura et al. 2005, 2007; Chater et al. 2011; Sakakibara et al. 2014; Eklund et al. 2015; Flores-Sandoval et al. 2015; Kato et al. 2015; Honkanen et al. 2016; Moody et al. 2018). To understand the hornwort biology, *Anthoceros agrestis* has recently been introduced as an experimental system and the first preliminary analyses of the nuclear genome sequence were also published (Szovenyi et al. 2015; Szovenyi 2016). However, tools for hornwort molecular study have not yet been developed.

One of the major innovations during terrestrialization by the first land plants was the transition from a haplobiontic to a diplobiontic life cycle in an algal ancestor (Kenrick 1994). The acquisition of this feature was a result of the evolution of the regulatory mechanisms governing developmental and physiological processes that led to the formation of a multicellular sporophyte. Similarly, the transition from a haploid- to a diploid-dominant alternation of generations must have been accompanied by radical changes in regulatory mechanisms, enabling large-scale alterations in the function and morphology of both generations. Genomic mechanisms underlying sporophyte development could have evolved de novo or by the partial or full transfer of preformed gametophytic programs to sporophyte generation (Nishiyama et al. 2003; Dolan 2009; Niklas and Kutschera 2009). Recent studies, primarily conducted in the model moss *P. patens*, have revealed details about the evolution of alternation of generations and embryogenesis. The crucial players for the moss alteration of the gametophyte in the sporophyte generation have been shown to belong to the homeodomain transcription factors (TFs) KNOX and BELL families (Sakakibara et al. 2008, 2013; Horst et al. 2016; Ortiz-Ramirez et al. 2017). Also important discoveries concerning the origin of an ancient genetic mechanism needed for plant fertility were found on *M. polymorpha.* In this liverwort, it has been shown that the MpRKD TF from the RWP-RK family is required to establish and/or maintain the egg cells quiescent in the absence of fertilization (Rovekamp et al. 2016) and control the formation of germ cells (Koi et al. 2016). Another example, MpBONOBO (MpBNB), a single member of the subfamily VIIIa of basic helix‒loop‒helix TFs, was shown to be responsible for developmental control of gametangiophore initial cells into mature gametangia. Moreover, MpBNB can functionally complement two orthologous *Arabidopsis thaliana BNB* genes that control pollen generative cell formation, suggesting that these TFs have been core regulators for reproductive development since the early stages of land plant evolution (Yamaoka et al. 2018). Likewise, the evolution of DUO POLLEN 1-type MYB TFs was shown to be a major event leading to the emergence and maintenance of sperm differentiation in the land plant lineage (Higo et al. 2018). The sparse taxon sampling among bryophytes for genomic and functional studies impedes our understanding of land plant evolution. Thus, more species for genome-scale sequencing and functional studies are required. However, plant species tend to have larger and more complex genomes than animals, which is often a challenging feature for genome sequencing and assembly. RNA sequencing represents an attractive and cost-efficient alternative to whole-genome sequencing, an approach that, in recent years, has revolutionized the genomics of model as well as non-model plant species (Matasci et al. 2014). RNA-seq enables the quantitative assessment of transcripts between different cells, tissues, organs, or organisms, but also is a powerful tool for the discovery of novel transcript species including long noncoding RNA, miRNA, siRNA, and other small RNA classes.

Our previous studies utilizing combined RNA-seq analyses of the transcriptome, small RNAs, and the degradome data from liverwort *Pellia endiviifolia* species B provided experimental evidence for target mRNA turnover by identified conserved and novel miRNAs. We showed the existence of common miRNAs between algae and land plants, suggesting that liverworts are at the base of the land plant evolutionary tree (Alaba et al. 2015). *P. endiviifolia* is a dioecious liverwort from the class Jungermanniopsida (Supplementary Data S1: Fig. S1–S2), one of three liverwort classes, which comprise simple thalloid and leafy clades accounting for over 80% of presently living liverwort taxa. Representatives of the genus *Pellia* are recognized as the most basal lineage of the simple thalloid liverworts with regard to many plesiomorphic features, such as the cuneate apical cell, a thallus without the midrib, a spherical capsule, and massive seta (Pacak et al.^1998^; Pacak and Szweykowska-Kulinska 2003; He-Nygren et al. 2006; Crandall-Stotler et al. 2009). In comparison with *M. polymorpha,* which belongs to the class Marchantiopsida comprising the complex thalloid liverworts, representatives of simple thalloid forms never produce specialized gametangiophores. Their antheridia and archegonia are born at the thallus surface (Supplementary Data S1: Fig. S2) (Schuster 1992; Paton 1999). In our previous work, we identified several genes expressed solely in the male or female reproductive organs of *P. endiviifolia* by utilizing the representational difference analysis of cDNA (RDA-cDNA) technique (Sierocka et al. 2011, 2014). To enhance our understanding of the genetic control governing *P. endiviifolia* male and female gametophyte development, RNA sequencing was performed on thalli collected from two sources: the first from the natural habitat during sex organ production and the second grown in axenic culture without sex organs. Distinct gene sets were identified as differentially regulated between male and female individuals in relation to different growth conditions, but differences in gene expression were also observed between plants depending on whether or not they were producing sex organs. Finally, we point out several genes as potential candidates for further functional studies to test their involvement in liverwort sexual reproduction.

## Materials and methods

### Plant material

Female and male thalli of *P. endiviifolia* species B producing sex organs (Herbarium number 40228 in POZW) were collected and cultured as previously described (Alaba et al. 2015), with the only difference being the year of collection (2011–2013). For RNA sequencing, four types of *Pellia* gametophytes were collected: (i) female thalli without archegonia cultivated in vitro (Fiv), (ii) female thalli-producing archegonia collected from the natural habitat (2011 and 2012 seasons) (Fng), (iii) male thalli with and without antheridia cultivated in vitro (Miv), and (iv) male thalli-producing antheridia collected from the natural habitat (2011 and 2012 seasons) (Mng) (Supplementary Data S1: Fig. S2). For real-time PCR experiments, the same types of *Pellia* thalli were used but from the 2013 season.

### RNA extraction and deep sequencing

Total RNA was isolated using a single-step method described by Chomczynski and Sacchi (1987), and modified as described by Pant et al. (2009). DNA was removed by digestion using RNase-free TURBO™DNase (Ambion^®^, Austin, TX, USA). The lack of genomic DNA contamination was confirmed by PCR using primers designed for the *P. endiviifolia SKP1* gene promoter sequence (accession number FJ266076; PSkp1_For: gctatactacatcatccagtt; PSkp1_Rev: aaacacaataactaccgcacg) (Supplementary Data S1: Fig. S3), ~ 150 ng of RNA as a template, and DreamTaq DNA polymerase (Thermo Fisher Scientific, Waltham, MA, USA). RNA integrity was checked on 1.2% agarose gels prior to and after the DNase digestion and measured with GeneTools image analysis software (Syngene Synoptics, Cambridge, UK). RNA isolation steps were performed multiple times for each thalli type. RNA isolates from each thalli type were next combined separately to generate the four RNA samples that met the criteria acceptable for plant RNA-seq (Quantification): total RNA mass ≥ 5 µg, concentration ≥ 150 ng/µL, r26S/18S ≥ 1, OD 260/280 ≥ 1.8, and OD 260/230 ≥ 1.8, and were used to prepare four cDNA libraries, each of which represented one type of thallus for one biological replicate. The RNA samples were shipped on dry ice to the Beijing Genome Institute (BGI, Beijing, China) for cDNA library preparation and sequencing in one 50SE lane on an Illumina HiSeq2000 system (Illumina, San Diego, CA, USA). Raw read sequences are available in the Short Read Archive database from the National Center for Biotechnology Information (NCBI, Bethesda, MD, USA) with the accession number (BioProject ID: PRJNA596103). Filtering of raw data was performed by BGI. Clean reads (clean data) were obtained by removing reads with adaptor sequences, reads with greater than 10% of unknown bases (*N*), and low-quality reads. Clean reads were mapped to reference sequences—de novo transcriptome data from *P. endiviifolia* (Alaba et al. 2015)—using SOAPaligner/SOAP2 (Li et al. 2009). No more than two mismatches were allowed in the alignment. For each RNA sample, more than 42 million total reads were obtained, where more than 99% were clean reads. Sequencing of all four samples reached the correct degree of saturation as well as the distribution of readings equated to reference transcriptome sequences was found to be statistically significant. From over 40 million clean reads from each tissue studied, around 90% were mapped to the *Pellia* transcriptome data (Supplementary Data S2: Table S1).

### Differential gene expression analysis

First, the expression level for each transcript was determined by the number of reads uniquely mapped to the specific unigene from the transcriptome data and the total number of uniquely mapped reads in the given tissue sample. Next, to normalize the transcript expression levels, we used the reads per kilobase per million reads (RPKM) method (Mortazavi et al. 2008) by the formula:

$${\text{RPKM}}\left( X \right)\, = \,\left( {10^{6} \, \times \,C} \right)/({\text{NL}}/10^{3} ).$$

Here, RPKM(*X*) is the expression of transcript *X*, *C* is the number of reads that uniquely aligned to transcript *X*, *N* is the total number of reads that are uniquely aligned to all transcripts, and *L* is the number of bases on transcript *X*. The RPKM method eliminated the influence of different gene lengths and sequencing levels on the calculation of gene expression. To identify DEGs between two-thalli types, the algorithm based on Poisson distribution model (Audic and Claverie 1997) was used with the following cut-off values: false discovery rate (FDR) ≤ 0.001 and log2fold change ≥ 2. Since our initial analysis was performed on sequencing results without replicates, we have further filtered the identified differentially expressed genes using edgeR tool by suppling 0.4 as the common BCV (square-root-dispersion) value (Robinson et al. 2010). Genes showing FDR < 0.05 were designated for subsequent analyses. The selected unigenes were used for blast searching with annotation against NCBI nonredundant sequence database, Kyoto Encyclopedia of Genes and Genomes (KEGG) protein database (Kanehisa and Goto 2000), *M. polymorpha* genome database MarpolBase (Bowman et al. 2017), 1000 plant project (1KP) project database (Matasci et al. 2014) using an *E* value cut-off < 10^−5^. Next, the distribution of gene functions was annotated using the Blast2GO program (Conesa and Götz 2008) to obtain the gene ontology (GO) functional classification.

### Real-time PCR analysis

First-strand cDNA synthesis was carried out using Oligo-dT_(18)_ (Life Technologies) and superscript III reverse transcriptase (Invitrogen, Waltham, MA, USA) according to the manufacturer’s instructions. Real-time PCR was performed using a 7900HT fast real-time PCR system and power SYBR green PCR master mix (Applied Biosystems, Waltham, MA, USA), 1 µL of cDNA, and gene-specific primers (200 nM each) in a final volume of 10 µL. The following thermal profile was applied for all real-time PCRs: 95 °C for 10 min; 40 cycles of 95 °C for 15 s, and 60 °C for 1 min. After each real-time PCR run, dissociation curve analyses were performed to confirm the primer specificity. The results were analyzed with SDS 2.2.3 software (Applied Biosystems). Ct values for all transcripts from two biological replicates were normalized to the *ACTIN1* (GenBank: DQ100290) Ct value. The relative expression level was calculated using the comparative ΔΔCt method. The primers that were used in this study are listed in Table S2 (Supplementary Data S2). Primers amplifying the fragment of the *PenB_TUA1* gene specifically expressed in male individuals (GenBank: HQ634388) were used in RT-PCR and RT-qPCR analysis as a marker for male-specific expression (Sierocka et al. 2011). Primers amplifying the fragment of *the PenB_MT2* gene specifically expressed in female individuals (GenBank: KF853594.1) were used in RT-PCR and RT-qPCR analysis as a marker of female-specific expression (Sierocka et al. 2014).

### RACE experiments

5′RACE for the identification of the transcription start sites and 3′RACE for the identification of possible open-reading frames for selected DEGs were carried out. RACE experiments were performed as previously described (Sierocka et al. 2011, 2014). The primer sequences used in the RACE experiments are shown in Table S2 (Supplementary Data S2).

### Sequence analysis

Database searches of the nucleotide and deduced amino acid sequences were performed using NCBI/GenBank/Blast searching (Altschul et al. 1990), the *M. polymorpha* genome database (Bowman et al. 2017), and Blast for 1000 Plant Transcriptomes on the 1KP project website (Matasci et al. 2014). The search for specific amino acid sequences was conducted using MotifScan (https://myhits.isb-sib.ch/cgi-bin/motif_scan), InterProScan (Jones et al. 2014), and SMART (Letunic et al. 2012).

### GenBank accession numbers

Sequences of full or partial cDNA sequences obtained after RACE experiments were submitted to the GenBank database under the accession numbers MN495995‒MN496037.

## Results

### Differentially expressed genes between four *P. endiviifolia* thalli types

The RNA sequencing using short reads on an Illumina HiSeq2000 system was performed on four RNA samples isolated from four *P. endiviifolia* thalli types collected in two seasons, as described in Sect. “Materials and methods”, in one biological replicate for each tissue type. For each sample, around 200 male or female individuals were collected. The types of tissue selected represented the set of male and female gametophytes for the study of differences in gene expression between the vegetative and reproductive stages of *Pellia* individuals during development.

**Fig. 1:**
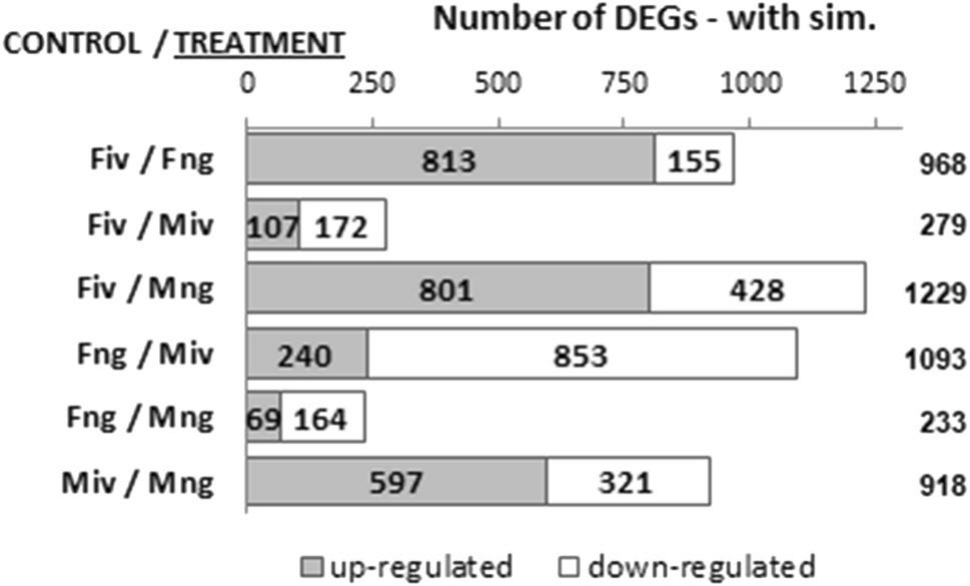
Chart listing statistics of the groups of differentially expressed genes between the two *P. endiviifolia* thalli comparison for DEGs showing similarity to sequences deposited in the public databases. The differentially expressed genes were identified with the criteria (twofold or more change and filtered using edgeR tool with FDR ≤ 0.05). In a pairwise comparison, the former one is considered as the control, and the latter one is considered as the treatment. *Fiv* female thalli grown in vitro, *Fng* female thalli with archegonia collected from natural habitat, *Miv* male thalli grown in vitro, *Mng* male thalli with antheridia collected from natural habitat

Out of 62,711 unigenes assessed in the *P. endiviifolia* transcriptome (Alaba et al. 2015), we identified 37,801 expressed in male gametophytes without antheridia grown in vitro, 44,316 in male gametophytes producing antheridia and collected from their natural habitat, 38,586 in female gametophytes without archegonia grown in vitro, and 43,898 in female gametophytes producing archegonia and collected from their natural habitat. Differences in gene expression were then obtained by pairwise comparison of the four libraries, which resulted in six lists of DEGs (Supplementary Data S3, Supplementary Data S1: Fig. S4a). Interestingly, over 50% of the genes identified in each two-thalli comparison showed no similarity to the sequences deposited in the public databases (Supplementary Data S1: Fig. S4b), even when they were used in a Blast search against the *M. polymorpha* genome and the 1KP project database. Thus, removing unannotated DEGs markedly decreased the number of identified genes in the two-thalli comparisons (Fig. 1). Nevertheless, the trend for all six lists remained the same: the fewest DEGs were observed whenever the male and female thalli were grown under the same conditions, and in the contrary, the highest DEG numbers were observed whenever the male and female thalli were grown in different conditions (Fig. 1, Supplementary Data S1: Fig. S4).

To display the number of transcripts expressed commonly and specifically for the two-thalli comparison, three Venn diagrams (Supplementary Data S1: Fig. S5) were generated. Two Venn charts were prepared to represent specifically expressed genes in female (Supplementary Data S1: Fig. S5a) and male (Supplementary Data S1: Fig. S5b) in connection with the developmental phase and growth conditions. A third graph (Supplementary Data S1: Fig. S5c) combines differential gene expression from all six lists obtained from our RNA-seq experiment.

Our previous studies utilizing the RDA-cDNA technique allowed us to identify several genes specifically expressed in male or female individuals of *P. endiviifolia* (Sierocka et al. 2011, 2014). Transcripts for four genes were also present in our RNA-seq data, and their expression pattern is consistent with our previous results (Supplementary Data S2: Table S3).

### Differentially expressed genes associated with *P. endiviifolia* reproductive phase of growth

In the process of DEG screening, we decided to narrow down our interest to genes whose expression in *Pellia* individuals producing sex organs is changed in comparison to the remaining thalli types. As a result, we identified 786 DEGs whose expression profile is associated with *Pellia* male individuals producing antheridia (Mng): 387 were up-regulated and 399 were down-regulated. In the case of genes whose expression is associated with *Pellia* female thalli-producing archegonia, we identified 516 DEGs, 391 up-regulated and 125 down-regulated (Fng) (Supplementary Data S2: Table S4; Supplementary Data S5). To further analyze the possible function of selected genes, we assessed their GO classifications. Based on the sequence similarity, 144 Mng and 101 Fng DEGs were categorized into 28 functional groups (Supplementary Data S1: Fig. S6). In each of the three main categories, the most abundant in DEGs were “metabolic process”, “cellular process”, and “response to stimulus” for biological processes; “cell”, “cell part”, and “organelle” for cellular components; and “catalytic activity” and “binding” for molecular function. The sequences were further evaluated using the KEGG database: 209 Mng and 141 Fng DEGs were categorized into 20 KEGG pathways, with the highest enrichment in metabolic pathways (Supplementary Data S1: Fig. S7). In this category, “biosynthesis of other secondary metabolites” and “carbohydrates metabolism”, were the largest categories. When we looked closer at the particular pathways, we noticed an enrichment in the “ribosome” pathway, and 14 male and 13 female DEGs with a similarity to ribosomal proteins accounted for over two-thirds of the KEGG “translation” category. As ribosomal proteins (RPs) are essential for protein synthesis, they play a crucial role in cell survival. In addition to this function, the phenotypes resulting from mutations in several different plant *RP* genes provided strong evidence for the regulatory function of RPs in plant developmental processes (Byrne 2009). For the *Pellia* male individuals producing antheridia and grown in the natural habitat, we selected gene transcripts with a similarity to RPs important for normal embryo development (L4, L18, L23) and leaf patterning (L5, L10a). The same processes are represented by RPs selected in *Pellia* female individuals producing archegonia and grown in the natural habitat (S6, L18e, L22, and S18, respectively).

### The homologs of *M. polymorpha* genes essential for sexual reproduction are expressed in *P. endiviifolia* gametophytes producing sex organs

When the first liverwort genome was released in 2017, we looked for *M. polymorpha* orthologs of the identified *P. endiviifolia* genes. We narrowed down our analysis to DEGs whose expression was up-regulated in *Pellia* male and female thalli-producing sex organs. Of the 387 genes up-regulated in the male individuals producing antheridia, only 141 showed similarity to known sequences deposited in the public databases. Of the 391 genes up-regulated in female individuals producing archegonia, only 153 showed similarity to known sequences deposited in the public databases. For the 148 *Pellia* male and 144 female-specifically expressed genes, *M. polymorpha* orthologs were found (Supplementary data S6 and S7, respectively). In previous transcriptomic studies published by Araki’s group, the transcriptional framework of *Marchantia* male gametogenesis was described (Higo et al. 2016). Thus, we next used the obtained *P. endiviifolia* gene list to look for *M. polymorpha* orthologs engaged in sperm cell production. Our analysis revealed that 37 *P. endiviifolia* genes that are specifically expressed in *Pellia* male gametophytes producing antheridia exhibit a similar expression pattern to the *M. polymorpha* orthologs (Supplementary Data S6). Six of these are orthologs of the *Marchantia* genes specifically expressed in antheridiophores, while the remaining 31 *Pellia* genes are orthologs of *Marchantia* genes that show antheridiophore-enriched expression in comparison to the expression observed in archegoniophores, according to MarpolBase (Bowman et al. 2017) (Supplementary Data S6). The most represented categories included genes encoding proteins with tetratricopeptide and Armadillo-like repeats, genes associated with oxidation–reduction processes and vesicle transport—four genes in each category (Table 1, selected examples).

**Table 1:** Selected examples of *M. polymorpha* orthologs of identified *P. endiviifolia* male and female-specifically expressed genes in gametophytes producing sex organs

*Pellia* gene ID	RPKM value (Mng/Miv/Fng/Fiv)	Blastx NCBI: Marchantia polymorpha (taxid:3197)	*E* value	Annotation	Tracking id MarpolBase	Length	Antheridiophore	Archegoniophore	Sporophyte	Sporeling (HATC)	
						nt	FPKM^a^	FPKM^a^	FPKM^a^	FPKM^a^	
Male specifically expressed											
Unigene13762	10,291,144/0,549,728/0,411,713/1,004,893	MARPO_0049s0007	9,00E−127	Ferric chelate reductase	Mapoly0049s0007.1	3529	6,58,892	2,86,858	3,30,731	0,534,337	
CL4820.Contig2	12,861,185/1,27,397/4,060,709/3,095,815	MARPO_0199s0021	2,00E−56	SNARE motif, subgroup Qc	Mapoly0199s0021.1	2394	33,7801	25,5893	25,6137	26,5067	
Unigene21096	13,153,349 /1,086,866/5,954,228/2,003,123	**MARPO_0014s0103**	**2,00E−97**	**Potassium ion transporter family protein**	**Mapoly0014s0103.1**	**4234**	**20,1357**	**0,0,986,339**	**0,33,508**	**0,101,249**	
		MARPO_0070s0079	2,00E−93	Potassium ion transporter family protein	Mapoly0070s0079.1	2675	21,7491	0,492,237	2,37,537	0,0,976,186	
CL132.Contig2	35,86,321/2,980,021/10,548,984/9,457,328	MARPO_0106s0013	8,00E−10	Tetratricopeptide repeat	Mapoly0106s0013.1	8318	1,26,183	0,171,887	0,596,877	0,62,306	
Female-specifically expressed											
Unigene8944	0,776,305/0,733,542/2,807,479/0,087,641	MARPO_0526s0002	9,00E−66	ABC-2 type transporter	Mapoly0526s0002.1	3114	0,344,105	1,94,363	79,0103	0,496,975	
Unigene23704	Nd/Nd/9,282,026/Nd	MARPO_0030s0123	8,00E−40	Sugar efflux transporter for intercellular exchange	Mapoly0030s0123.1	1611	0,784,167	4,31,432	29,2534	0	
CL10631.Contig1	Nd/0,099,671/Nd/43,668,835	**MARPO_0040s0034**	1,00E−31	Chitinase class I	**Mapoly0040s0034.1**	**1632**	**0,0,123,774**	**65,4722**	**0,0,534,328**	**0,0,635,778**	
Unigene24732	Nd/Nd/0,101,221/36,34,404	**MARPO_0040s0034**	4,00E−39	Chitinase class I	**Mapoly0040s0034.1**	**1632**	**0,0,123,774**	**65,4722**	**0,0,534,328**	**0,0,635,778**	

^a^FPKM values of *M. polymorpha* genes from the data published by Bowman et al. 2017. In bold, *M. polymorpha* genes are marked which expression is antheridiophore- or archegoniophore-specific

We also used the MarpolBase transcriptomic data to check the expression profiles of *Marchantia* orthologs for the identified *P. endiviifolia* genes specifically expressed in female gametophytes producing archegonia. We found 50 *Pellia* genes for which *Marchantia* orthologs exhibit a similar expression pattern. Three of them display an archegoniophore-specific expression profile, while the remaining *Marchantia* orthologs show archegoniophore-enriched expression when compared to their expression in antheridiophores (Supplementary Data S7). The most represented functional categories included genes encoding different transmembrane transporters (eight genes), glycosyl hydrolases, and transferases (five genes for each category) (Table 1, selected examples).

In summary, after a comparison of our data with the *M. polymorpha* gene expression profiles, we found similarities in specific or enriched gene expression during the reproductive phase of growth for both liverwort species. The common expression of 87 selected genes suggests a common mechanism governing sex organ development in representatives of the two distinct liverwort classes.

### Quantification of the selected DEGs in qPCR assay

To identify genes showing the highest differences in expression between the antheridia-producing male thalli and archegonia-producing female thalli of *P. endiviifolia*, we used the formula of log2 (Fng/Mng) ≥ 10. As a result, we selected 72 DEGs, 10 with ≥ tenfold higher expression in the male thalli with antheridia and 62 with ≥ tenfold higher expression in the female thalli with archegonia, both grown in their natural habitat (Supplementary Data S8). In addition, the 72 DEGs were juxtaposed with the expression level in the male and female thalli from the vegetative phase of growth from in vitro culture. This analysis showed that out of the ten genes up-regulated in sperm-producing male thalli, eight are also expressed during the vegetative phase of growth. In turn, out of the 62 up-regulated gene transcripts in the archegonia-producing female thalli, 46 are also expressed during the vegetative phase of growth. The most enriched DEGs belong to RNA- or DNA-binding protein families (*pen_RRM-I, pen_ARR-like*, *pen_MYB1*, *pen_SPL1*), LRR receptor-like kinases (*pen_RLK10.1*, *pen_RLK10.2*, *pen_RLK10.3*), ubiquitin protein ligases (*pen_UPL2.1*, *pen_UPL1.1*, *pen_UPL2.2*), and serine/threonine protein phosphatases (*pen_BSL2-like*, *pen_PP2C.1*, *pen_PP2C.2*). Thirty-five DEGs showed no similarity to sequences from the public databases; the lengths of these transcripts range from ~ 250 to ~ 600 nt with no putative open-reading frames identified (Supplementary Data S8).

**Fig. 2:**
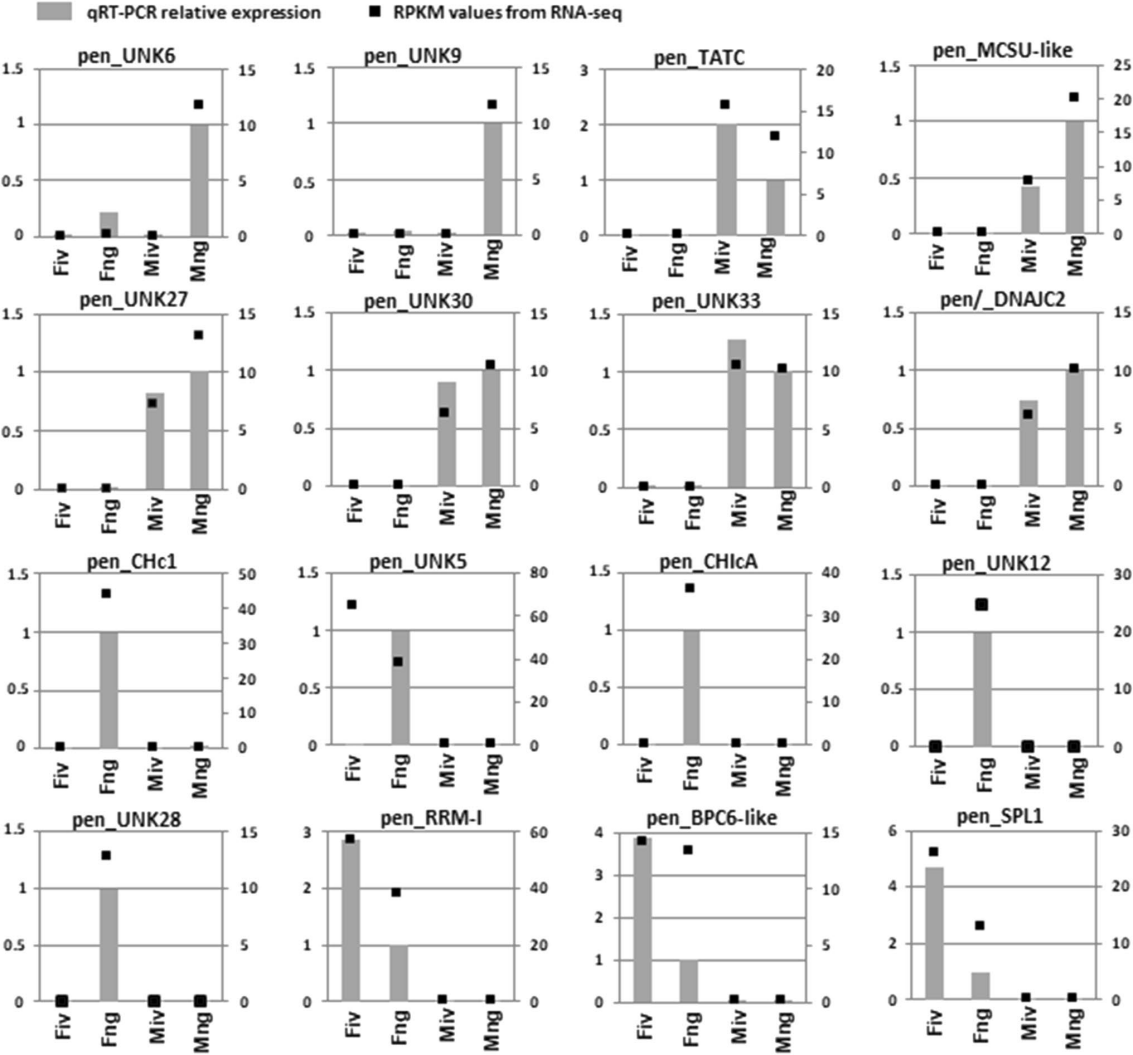
Comparison of gene expression data obtained by RNA-seq and RT-qPCR. Expression levels of selected genes in four types of *Pellia* thalli are shown. *Fiv* female thalli grown in vitro, *Fng* female thalli with archegonia collected from natural habitat, *Miv* male thalli grown in vitro, *Mng* male thalli with antheridia collected from natural habitat. *Y*-axis on the left side of graphs shows the scale for qPCR values (grey boxes) normalized against *ACTIN1* gene from two biological replicates. *Y*-axis on the right side of graphs shows the scale for RPKM values (black squares) from NGS

To validate the RNA-seq data of male- or female-specific patterns of gene expression, RT-qPCR analysis was performed on 72 selected DEGs for the four types of *Pellia* thalli, the same as for RNA-seq, but on new material collected in the 2013 season. The qPCR results confirmed the calculations for RPKM values from the RNA-seq experiment for 54 out of 72 selected transcripts (Fig. 2, Supplementary Data S1: Fig. S8) with minor discrepancies. Eight out of ten genes showed male-specific expression in both of the male thalli tested. The remaining two, *pen_UNK6* and *pen_UNK9*, as in the data from RNA-seq, showed specific expression only in the male thalli-producing antheridia and grown in the natural habitat (Fig. 2).

Forty-six DEGs selected as female-specifically expressed from the RNA-seq data also showed female-specific expression in the qPCR analysis. Five genes, *pen_CHc1*, *pen_UNK5*, *pen_CHIcA*, *pen_UNK12*, and *pen_UNK28* (Fig. 2), are expressed only in the female thalli-producing archegonia and grown in the natural habitat. The remaining 39 are expressed in both female thalli tested, regardless of whether they produce archegonia (Fig. 2, Supplementary Data S1: Fig. S8). The observed differences in gene expression between male and female *P. endiviifolia* individuals may reflect the specific gene sets responsible for the execution of developmental pathways, leading to the development of antheridia-producing male thalli and archegonia-producing female thalli.

In the case of the 18 DEGs, four were revealed to be false positives according to real-time PCR. The remaining 14 showed no amplification in any of the four types of thalli used for the study, even though several primer pairs were designed. None of those 14 DEGs showed similarity to sequences deposited in the publicly available databases, even when different Blast algorithms were used. It should be noted that all 14 sequences were identified in female individuals collected from their natural habitat in the 2011 season, whereas for qPCR analysis, female individuals collected in the 2013 season were used. Therefore, it cannot be ruled out that some environmental conditions determining the expression of those DEGs differed between the 2011 and 2013 seasons, which could explain the observed results.

### Molecular characterization of full-length transcripts of selected DEGs

Many of the gene transcripts validated by qPCR showed no similarity to the sequences from the public databases, even when the *M. polymorpha* genome database was searched. Most of them did not exceed 600 nt in length. Moreover, in some, the potential start or end of the open-reading frame was computationally identified, which may indicate that these transcripts are, in fact, 5′ or 3′ UTR parts of the mature mRNAs. To determine the mRNA end for these transcripts, RACE experiments were performed. Additionally, for all DEGs validated by qPCR, 5′RACE was performed to determine their transcription start sites (TSS). The results of RACE analysis are summarized in Table S5 (Supplementary Data S2). Only in four cases (genes: *pen_UNK3*, *pen_RRM-I*, *pen_UNK18*, *pen_UNK19*), we were able to determine the coding sequence (CDS) after RACE experiment. This analysis also unexpectedly revealed that 21 out of 46 female-specific DEGs validated in qPCR, in fact, originate from seven gene transcripts, which gave us a final total of 30 specifically female-expressed genes. For some of the genes, different mRNA isoforms were identified that arise from alternative splicing events (genes: *pen_RRM-I*, *pen_UNK18*, *pen_AGD5-like1*, *pen_LRR-RLK1*) or the selection of different polyadenylation sites (e.g., *pen_RRM-I*, *pen_ARR-like*, *pen_YIPPEE-like*, *pen_UNK33*, *pen_TUA2*). Additionally, in the case of *pen_UNK3*, *pen_UNK18*, and *pen_AGD5-like1* genes, alternative splicing results in two mRNA isoforms with different protein-coding regions. For the *pen_LRR-RLK1* gene, alternative splicing occurs within 3′UTR and does not change the CDS.

### Expression of selected DEGs in archegonia and antheridia

**Fig. 3:**
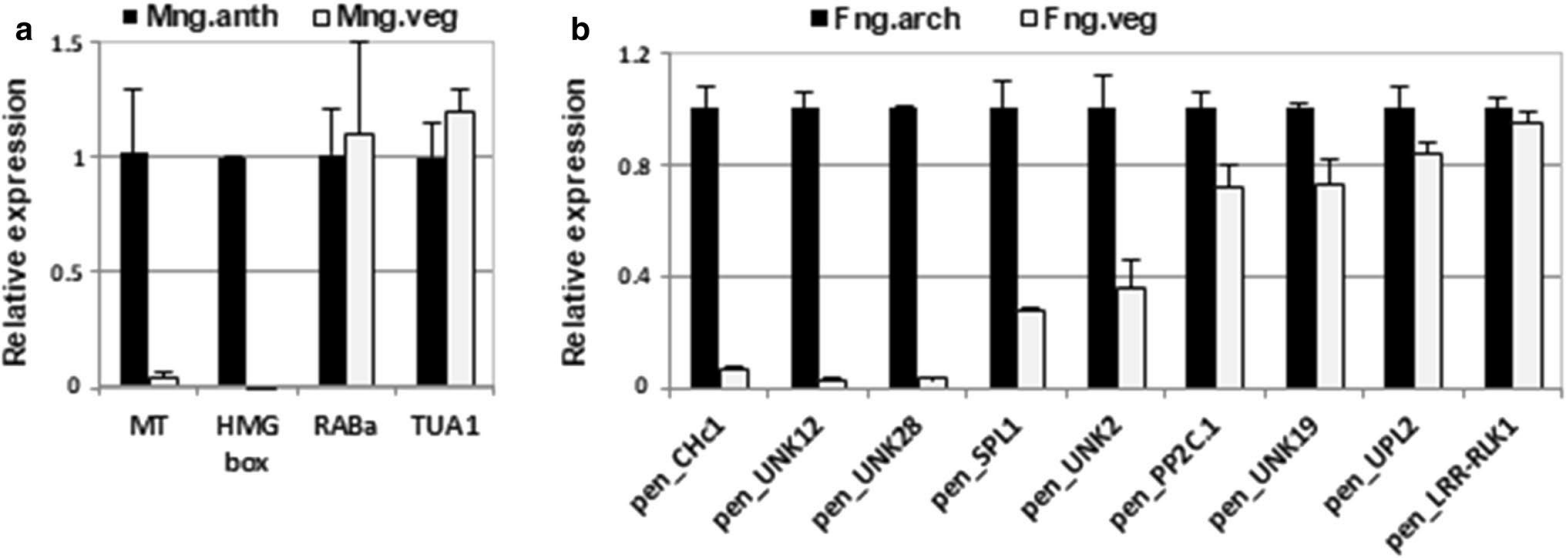
Several *P. endiviifolia* genes expression is correlated with antheridia or archegonia production. Quantitative RT-qPCR analyses of the (**a**) male-specifically expressed RDA-cDNA/RNA-seq identified genes and (**b**) the female-specifically expressed RNA-seq identified genes with different transcript levels in the vegetative and sex organs of male and female gametophytes grown in natural habitat, respectively. **a** The relative expression of each gene was compared with antheridia (Mng.anth) isolated from the vegetative parts of male thalli (Mng.veg). **b** The relative expression of each gene was compared with archegonia-bearing region (Fng.arch) isolated from the vegetative parts of female thalli (Fng.veg). All transcript levels were normalized against *ACTIN1*. Error bars indicate mean ± SD; *n* = 2

In the RNA-seq and qPCR analysis, whole male and female thalli-bearing sex organs were used, where most of the tissue is responsible for the vegetative growth and function. Antheridia, situated in 1‒2 irregular rows at the dorsal part of the male gametophyte, are 200 µm in diameter. Archegonia, 8‒12 shielded by the cylindrical involucre, are grouped in a zone 3 mm in diameter (Schuster 1992; Paton 1999). To investigate whether the elevated expression in the male or female thalli corresponded with antheridia- or archegonia-specific expression, a quantitative real-time PCR experiment was performed on RNA isolated from the vegetative and reproductive parts of the male and female gametophytes separately from vegetative ones. Approximately 500 antheridia from 70 male gametophytes were dissected from the vegetative part of thalli. The archegonia-bearing region, together with the involucre (3 mm × 3 mm in size), was dissected as previously described (Sierocka et al. 2014). RNA preparation and subsequent qPCR analysis were carried out separately for both generative and vegetative samples. Of the ten genes selected as specifically expressed in male, none showed enriched accumulation within the antheridia-isolated RNA (Mng.anth) (Supplementary Data S1: Fig. S9a). In this study, we also included the four male-specific genes identified by the RDA-cDNA approach, as previously described (Sierocka et al. 2011) from which two are present in our RNA-seq data (Supplementary Data S2: Table S3). Interestingly, two genes, *PenB_MT* and *PenB_HMGbox*, exhibit preferential expression in antheridia in comparison to the vegetative parts of the male thalli (Fig. 3a). We identified the homolog of *Pellia* HMG protein in the *Marchantia* genome and checked its expression profile in the available RNA-seq data. Strikingly, *Mapoly0031s0059.1* was specifically expressed in antheridiophores (Supplementary Data S2: Table S6). This result strengthens the observations of our previous study, in which we proposed that both *PenB_MT* and *PenB_HMGbox* are important players during antheridia development in the natural environment, as their expression was shown only in male gametophytes producing antheridia and grown in their natural habitat (Sierocka et al. 2011).

From 30 expressed genes selected as female-specific, three showed more than tenfold higher expression level in the archegonia-producing area of the female thalli (Fng.arch) in comparison to the female vegetative parts of thalli (Fng.veg) (Fig. 3b). These are the *pen_UNK12* and *pen_UNK28* genes, which showed no similarity to sequences from the public databases, and the *pen_CHc1* gene, with similarity to genes encoding plant class I chitinases. Another two cases, the *pen_SPL1* gene-encoding plant-specific SQUAMOSA promoter-binding protein-like (SPL) TF and the *pen_UNK2* gene with no similarity to known sequences, showed an approximately threefold higher expression level in the archegonia-bearing section when compared with the vegetative parts of the female thalli. Importantly, the two genes encoding proteins have their orthologs in *M. polymorpha.* Both of them exhibit a similar expression pattern to their *Pellia* counterparts (Supplementary Data S2: Table S6). *MpSPL1* (*Mapoly0014s0224.1*) shows the highest expression in archegoniophores, although it also has a high expression level in antheridiophores and sporophytes. The *Mapoly0040s0034.1* gene-encoding chitinase is specifically expressed in archegoniophores. Recently, a paper describing *MpSPL2* gene function was released, which in phylogenetic context is the closest paralog of *MpSPL1* (Tsuzuki et al. 2019)*.* It was shown that *MpSPL2* may play a role in promotion of reproductive transition in response to inductive light conditions as it is necessary for proper development of reproductive gametangiophores (Tsuzuki et al. 2019). Interestingly, *MpSPL2* exhibits a similar expression profile as *MpSPL1* what may suggest that both *SPL* genes are important for *Marchantia* reproductive phase stimulation.

Overall, from 30 gene transcripts selected as specifically female-expressed, we have validated that five are enriched in the archegonia-bearing region of *P. endiviifolia* female thalli. The rest of the transcripts showed a comparable level of expression in both types of female tissues or higher expression in the vegetative parts of the female thalli (Supplementary Data S1: Fig. S9b, selected examples). The observed expression of gene transcription pattern for the two male and five female genes may indicate their direct involvement in the development of *P. endiviifolia* sex organs. However, more genes need to be tested with a similar approach to get a more comprehensive view of the essential gene set responsible for sexual reproductive success of *P. endiviifolia.* Further functional studies will reveal their true involvement in liverwort sexual reproduction processes.

## Discussion

In the present study, we explored the transcriptional landscape of the *P. endiviifolia* male and female gametophytes with a distinction between the vegetative and reproductive developmental phases of growth in combination with two growth conditions. To our best knowledge, this is the first report on the gene expression profile in dioecious representatives of simple thalloid liverworts. Whenever the genes expressed in *Pellia* thalli grown in axenic culture were compared to genes expressed in those grown in their natural habitat, regardless of the sex, the differentially expressed gene number was more than double when compared to the genes expressed between the *Pellia* thalli grown in the same conditions, showing that the growth conditions have a significant impact on the regulation of gene expression. Additionally, during culture of *P. endiviifolia*, we could not obtain archegonia, and antheridia appear rarely, demonstrating that the axenic conditions do not resemble those of natural environments.

From the obtained data, we determined the transcriptional changes in the sex organ-producing *Pellia* individuals, which comprise ~ 1.25% genes associated with antheridia-producing *Pellia* male thalli grown in the natural habitat (Mng) and 0.82% genes associated with archegonia-producing *Pellia* female thalli grown in the natural habitat (Fng) in the context of *Pellia* transcriptome data. Importantly, over 50% of the DEGs from the four libraries did not show any similarity to sequences deposited in the public databases, including the closest relative with sequenced genome, *M. polymorpha.* In fact, *Marchantia* genome annotation also revealed that, from 19,138 nuclear encoded protein-coding genes, 5821 lack any annotation (Bowman et al. 2017). This set of transcripts may include novel species- or clade-specific transcripts as lineage-specific and species-specific genes have also been identified in other plant species like *A. thaliana* (Lin et al. 2010), *Oryza sativa* (Campbell et al. 2007), *Solanum* spp. (Rensink et al. 2005), and legumes (Graham et al. 2004; Schmutz et al. 2010). On the other hand, most of the putative orphan DEGs which we identified are < 600 nt in length, and in the present results, we have shown that, in some cases, the RNA-seq derived transcripts are only a fragment of the full-length mRNAs, as shown by RACE experiments. Thus, it cannot be ruled out that many of the DEGs which we selected are partial fragments of mature transcripts. Taking into consideration that in our approach we used no biological replicates for RNA sequencing, the results presented here can serve as preliminary indication of differential gene expression and require independent confirmation in further studies.

Among the DEGs selected as related to the sex organ-producing *Pellia* individuals, only about 13% of transcripts were categorized into five main KEGG pathways. Within the metabolism pathways, the great majority were related to biosynthesis of other secondary metabolites together with carbohydrate metabolism. This result is consistent with many reports concerning phytochemical studies of bryophytes that show that liverworts are plants with enormous chemical diversity and a rich array of secondary metabolites, mainly terpenoids and aromatic compounds isolated from *P. endiviifolia* among others (Asakawa et al. 2013; Ludwiczuk and Asakawa 2014). On the basis of several studies, it has been suggested that the accumulation of secondary compounds can reduce UV penetration and damage to potential targets (Arroniz-Crespo et al. 2004; Otero et al. 2008; Martinez-Abaigar and Nunez-Olivera 2011; Fabon et al. 2012). KEGG analysis has shown enrichment of different metabolic pathways in both male and female *P. endiviifolia* gametophytes. However, in many metabolic categories, the number of DEGs was twice as high in the antheridia-producing male gametophytes in comparison to archegonia-producing female gametophytes. The reason for these differences might be disparities in the location of these two sex organs on the thalli. Antheridia are distributed at the plant surface and are visible as clear nodules, whereas archegonia are shielded by cylindrical involucre (Schuster 1992; Paton 1999). Thus, the higher enrichment in metabolic pathways in male gametophytes may serve to protect the more exposed antheridia against UV radiation. Second, the most enriched KEGG pathway in selected DEGs was genetic information processing, in which translation was the most enriched category with an almost equal number of DEGs between *Pellia* male thalli-producing antheridia and female thalli-producing archegonia. Over 90% of this category accounted for differentially expressed genes annotated as ribosomal proteins. Several studies describing mutation in mammal and plant genes encoding ribosomal proteins and ribosome assembly factors revealed some new facts about their fundamental role in many developmental processes other than mRNA translation. In *Arabidopsis* ribosomal proteins, mutants share developmental abnormalities such as reduced shoot growth, reduced cell proliferation, and increased leaf cells ploidy, or are characterized by more severe phenotypes, such as embryo lethality (reviewed in Byrne 2009). Among the DEGs selected from our *Pellia* RNA-seq data, there are several with annotations to known plant ribosomal proteins that are engaged in the flowering process. It is possible that differential expression of the identified *P. endiviifolia* genes encoding ribosomal proteins reflects the specific requirements for specialized translation machinery during germ cell specification and functional gamete formation in this plant species.

As expected, because of the phylogenetic relationship, we identified a set of genes with common expression profile that show preferential or specific expression during sex organ development for the representatives of two liverwort lineages, simple thalloid *P. endiviifolia* and complex thalloid *M. polymorpha*. Some of the male-specific expressed genes are responsible for intracellular vesicle trafficking. In all bryophytes, the process of antheridia development involves several divisions of dedifferentiated epidermal cells. Next, spermatids undergo a dynamic morphogenetic process called spermiogenesis which leads to flagellated sperm cell formation. The machinery controlling this process needs to produce proteins which further are directed to appropriate cell compartment to allow successful cellular differentiation and mature antheridia production. Thus, the identified *Pellia* genes might be important factors for liverworts antheridia development. Other groups of genes overrepresented in the *Pellia* male gametophytes producing antheridia included genes associated with oxidation–reduction processes. In flowering plants, the entire process of sexual reproduction, starting from gametogenesis through pollen/embryo sac growth, and up to double-fertilization, is under strict control of redox-mediated signaling (reviewed in Traverso et al. 2013; Zinta et al. 2016). For bryophytes studies, recently, it was shown that the loss of activity of the MpTCP1 TF affects the expression of several groups of enzymes involved in reactive oxygen species metabolism. In a consequence, the misbalanced redox signaling pathways cause archegoniophore cells over proliferation and abnormal archegoniophores bearing secondary archegoniophores production (Busch et al. 2019). Together, these data suggest that redox regulation network is important for coordination of developmental processes already in early diverging land plants.

In turn, in *Pellia* female individuals producing archegonia, the highest number of genes belongs to transmembrane transporters with two ABC transporters and two sugar efflux transporters. The proper development of archegonia depends on appropriate regulation of the size and shape of every cell after each of several cell division cycles followed by cell differentiation. During that process, different transporters are needed to supply the dividing cells with necessary nutrients, but are also responsible for transporting signal molecules to orchestrate the proper archegonia development. Many ABC transporters have been reported to have important function in pollen formation (reviewed in Zhao et al. 2016). Recently, it was additionally shown that *Arabidopsis* ABCG1 and ABCG16 transporters may be required to maintain auxin homeostasis in pistils what is crucial for proper pollen tube growth (Liu et al. 2020). Whether the ABC transporters are important for hormone/nutrient homeostasis maintenance in the liverworts female reproductive organs is a matter of future investigations.

Current studies in *M. polymorpha* have identified conserved TFs regulating several crucial steps in the sexual reproduction process of this liverwort. These include *FEMALE GAMETOPHYTE MYB* (Mp*FGMYB*) for female gametophyte development, Mp*BNB* for gamete progenitor cell specification, Mp*DUO1* for sperm differentiation, and members of the RWP-RK domain family for female gamete formation (Higo et al. 2018; Yamaoka et al. 2018; Hisanaga et al. 2019). However, none of the *Pellia* genes identified in our analysis is an ortholog of the gene governing programs of sperm and eggs development described recently in *Marchantia*.

Of the 72 selected genes with the highest expression changes between *P. endiviifolia* male and female thalli in the reproductive phase of growth, over 75% (54 DEGs) of these were verified by RT-qPCR, 16.6% (14 DEGs) showed no amplification products, and 5.5% (4 DEGs) were false positives. Gonzales and Joly (2013) published a paper concerning the impact of the RNA-seq approach on false-positive rates in differential gene expression detection. They showed that the choice of single-end instead of paired-end sequences produced higher false-positive rate than reducing the length of sequence reads from 100 to 50 bp. In the present results, we used 50 bp single-end sequencing without knowledge about the bias that might result from this approach. Having *P. endiviifolia* transcriptome data, we decided to use 50SE sequencing for the differential gene expression analysis. In our study, we also did not include any biological replicates. Such replicates are mandatory to ensure the valid biological interpretation of results, due to individual sample variation. However, we did ensure specimen diversity due to the use of 200 individual thalli per sample, as in our previous successful study reporting *Pellia* small RNA (Alaba et al. 2015). Using RT-qPCR, we confirmed the up-regulation of 10 genes in the male thalli-producing antheridia and 30 genes in the female thalli-producing archegonia. The RNA-seq selected genes, together with the ones previously identified in the RDA-cDNA experiment (Sierocka et al. 2011), were used to test their expression on RNA isolated from antheridia and archegonia separately from the vegetative parts of *Pellia* thalli. From 40 DEGs selected after the RNA-seq experiments, only five female-specific DEGs showed enriched expression in archegonia. For two *P. endiviifolia* genes encoding SPL TF and class I chitinase, we have identified their *Marchantia* orthologs and, surprisingly, both *Marchantia* genes also display specific or enriched expression in the female reproductive organs. Interestingly, the SPL proteins from both liverworts group together with *Arabidopsis* SPL8 protein (unpublished data), which is crucial for proper pollen sac development (Unte et al. 2003) and regulates gynoecium differential patterning (Xing et al. 2013). Furthermore, *MpSPL1* gene exhibits similar expression pattern as another representative of *Marchantia SPL* gene family, *MpSPL2,* which recently was shown to be necessary for proper development of reproductive branches (Tsuzuki et al. 2019). Taking together, this information indicates the *pen_SPL1* and *MpSPL1* genes as very interesting examples for further functional studies.

Of 14 selected genes, ten from RNA-seq and four from RDA-cDNA experiments (Sierocka et al. 2011), only two *Pellia* male-specific expressed genes showed enriched expression in antheridia. For these two genes, we found a *Marchantia* ortholog only for the *PenB_HMG-box* gene, whose antheridiophore-specific expression profile in *Marchantia* positively correlates with the expression profile which we observed in *P. endiviifolia*. As there are no available data about the function of the *Marchantia* genes selected in our study, it is not possible to indicate similarities or differences between the *SPL* and *HMGbox* gene families from the most ancient living land plants and highly diversified vascular plants. Further detailed analyses of the biological and developmental functions of the identified differentially expressed genes will be a matter for further investigation.

## Conclusions

In the present study, we have shown differences in gene expression profiles between male and female gametophytes of the simple thalloid liverwort *P. endiviifolia.* Our data provide the first look at the transcriptional landscape of a representative from the most basal lineage of the Jungermanniopsida class. A comparison of the obtained data with the first sequenced liverwort genome resulted in the identification of a group of genes exhibiting similar expression patterns between members of the two distinct liverworts’ body plans. Finally, we point out several genes as candidates for functional studies to test their involvement in liverworts’ sexual reproduction.

### *Author contribution statement*

IS and ZSK conceived and designed research. IS performed the experiments. SA and WMK analyzed RNA-seq data. IS and ZSK wrote the manuscript. AJ and WMK made critical comments on the manuscript.

## Electronic supplementary material

Below is the link to the electronic supplementary material.

Supplementary file1 (PDF 1654 kb)

Supplementary file2 (PDF 1154 kb)

Supplementary file3 (XLSX 1768 kb)

Supplementary file4 (XLSX 262 kb)

Supplementary file5 (XLSX 191 kb)

Supplementary file6 (XLSX 128 kb)

Supplementary file7 (XLSX 130 kb)

Supplementary file8 (XLSX 44 kb)

## Abbreviations

1KP: 1000 Plant project
DEGs: Differentially expressed genes
Fng/Mng: Female/male thalli-producing archegonia grown in natural habitat
RDA-cDNA: Representational difference analysis of cDNA
SPL1: SQUAMOSA promoter-binding protein-like
TF: Transcription factor
