# Supplementary material for: The identification of differentially expressed genes in male and female gametophytes of simple thalloid liverwort Pellia endiviifolia sp. B using an RNA-seq approach
Source: Planta. 2020 Jul 15;252(2):21. doi: 10.1007/s00425-020-03424-z (PMC7363739; doi:10.1007/s00425-020-03424-z)
Supplement: Supplementary file 1 — Supplementary file1 (PDF 1654 kb) [file 425_2020_3424_MOESM1_ESM.pdf]

Supporting Data S1: Figures S1 – S9

The identification of differentially expressed genes in male and female gametophytes of simple thalloid liverwort *Pellia endiviifolia* sp. B using an RNA-seq approach

Planta

Izabela Sierocka[1]*, Sylwia Alaba[2], Artur Jarmolowski[1], Wojciech M Karlowski[2], Zofia Szweykowska-Kulinska[1]

[1] Department of Gene Expression, [2] Department of Computational Biology, Institute of Molecular Biology and Biotechnology, Faculty of Biology, Adam Mickiewicz University, Poznan, Uniwersytetu Poznanskiego 6, 61-614 Poznan, Poland

*corresponding author: Izabela Sierocka, email: izapaste@amu.edu.pl

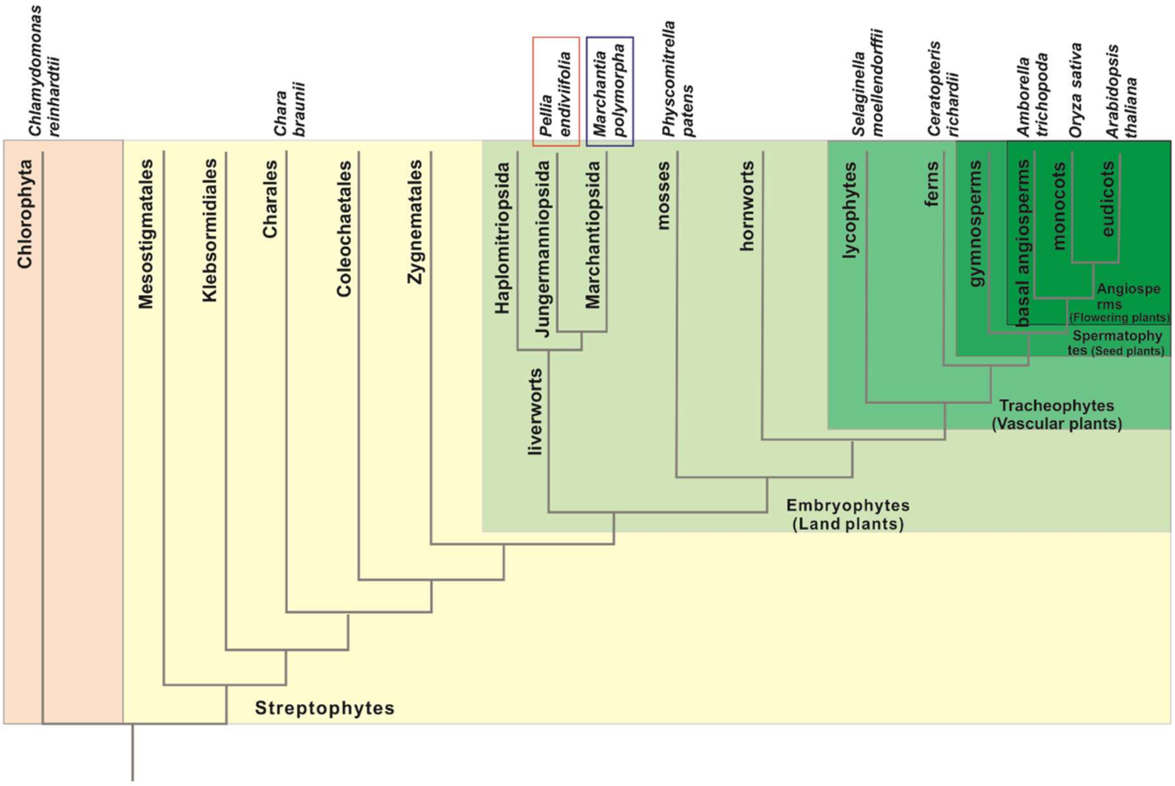

Fig. S1 Schematic evolutionary tree of Viridiplantae with marked phylogenetic position of *P. endiviifolia* and *M. polymorpha*. A simplified phylogenetic tree showing the evolutionary relationships among major lineages of the Viridiplantae based on Morris et al. study (2018).

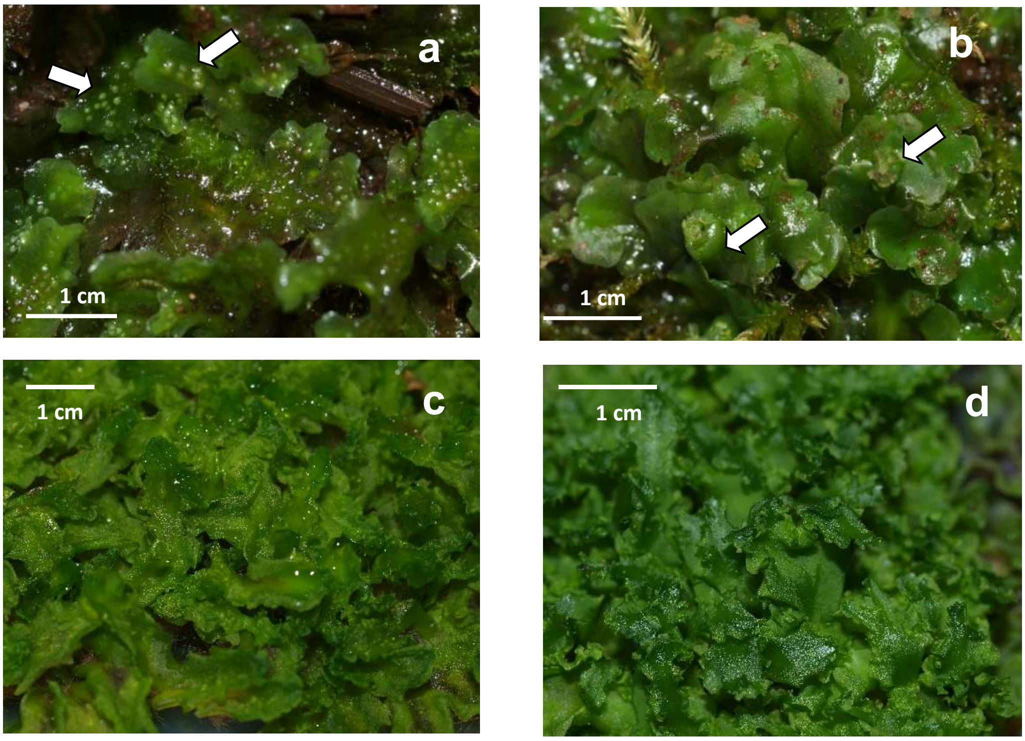

Fig. S2 Male (**a**) and female (**b**) thalli of the liverwort *Pellia endiviifolia* sp B grown in the natural habitat in Kopanina, Poznan, Poland and male (**c**) and female (**d**) thalli grown in *in vitro* culture (Konica Minolta Dynax5D). (**a**) The arrows point to irregular rows of antheridia on the male gamethophytes. The numerous antheridia are born dorsaly at the thallus surface in 1 – 2 irregular rows along the midrib. A characteristic feature of the genus *Pellia* is the secondary collapse of the antheridia into small chambers under the layer of surface cells of the thallus. A single superficial cell situated close to the thallus apical region acts as an atheridial initial. The spermatozoid developmet in essentially similar to that described in *Marchantia,* with only differences in detail of the multilayered structure and microtubule ribbon. (**b**) The arrows point to involucre sheltering the archegonia on the female gametophytes. The archegonia occur in groups 10 – 12 enclosed in a tubular, laterally flattened involucre along the dorsal mid-line of the thallus. In *P. endiviifolia* groups of archegonia lie in slightly raised transverse ridge of tissue called receptacle. A single superficial thallus cell enlarges outside and acts as an archegonial initial. The archegonium developmet in essentially similar to that described in *Marchantia,* with only difference that *Pellia* archegonium is more elongated due to variable number of the primary neck canal cell divisions (Schuster 1992; Paton 1999; Pandey et al. 2016).

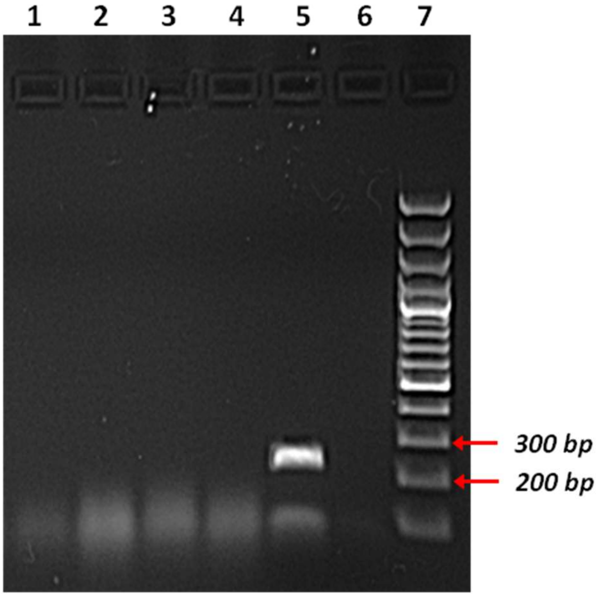

Fig. S3 The lack of genomic DNA contamination in *P. endiviifolia* RNA samples prepared for RNA sequencing. Agarose gel electrophoresis of PCR product using primers designed for *P. endiviifolia SKP1* gene promoter sequence on ~150 ng of RNA isolated for RNA sequencing from male thalli grown *in vitro* (lane 1), male thalli collected in the natural habitat (lane 2), female thalli grown *in vitro* (lane 3) and female thalli collected in the natural habitat (lane 4). The PCR positive control reaction with genomic DNA isolated from male thalli grown *in vitro* is shown in lane 5 and reaction without template is shown in lane 6. The 100 bp ladder is presented in lane 7.

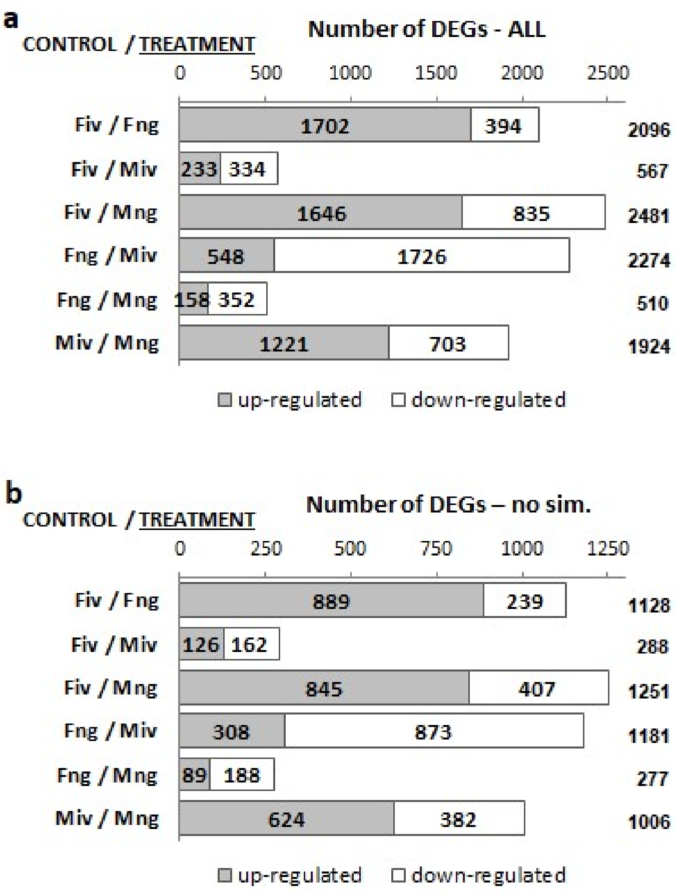

Fig. S4 Charts listing statistics of the groups of differentially expressed genes between the two *P. endiviifolia* thalli comparison: in (**a**) the numbers for all DEGs are presented, in (**b**) the numbers of DEGs showing no similarity to sequences deposited in public databases are presented. The differentially expressed genes were identified with the criteria (two fold or more change and FDR ≤ 0.05). In a pairwise comparison, the former one is considered as the control, and the latter one is considered as the treatment; Fiv – female thalli grown *in vitro*, Fng – female thalli with archegonia collected from natural habitat, Miv – male thalli grown *in vitro*, Mng – male thalli with antheridia collected from natural habitat.

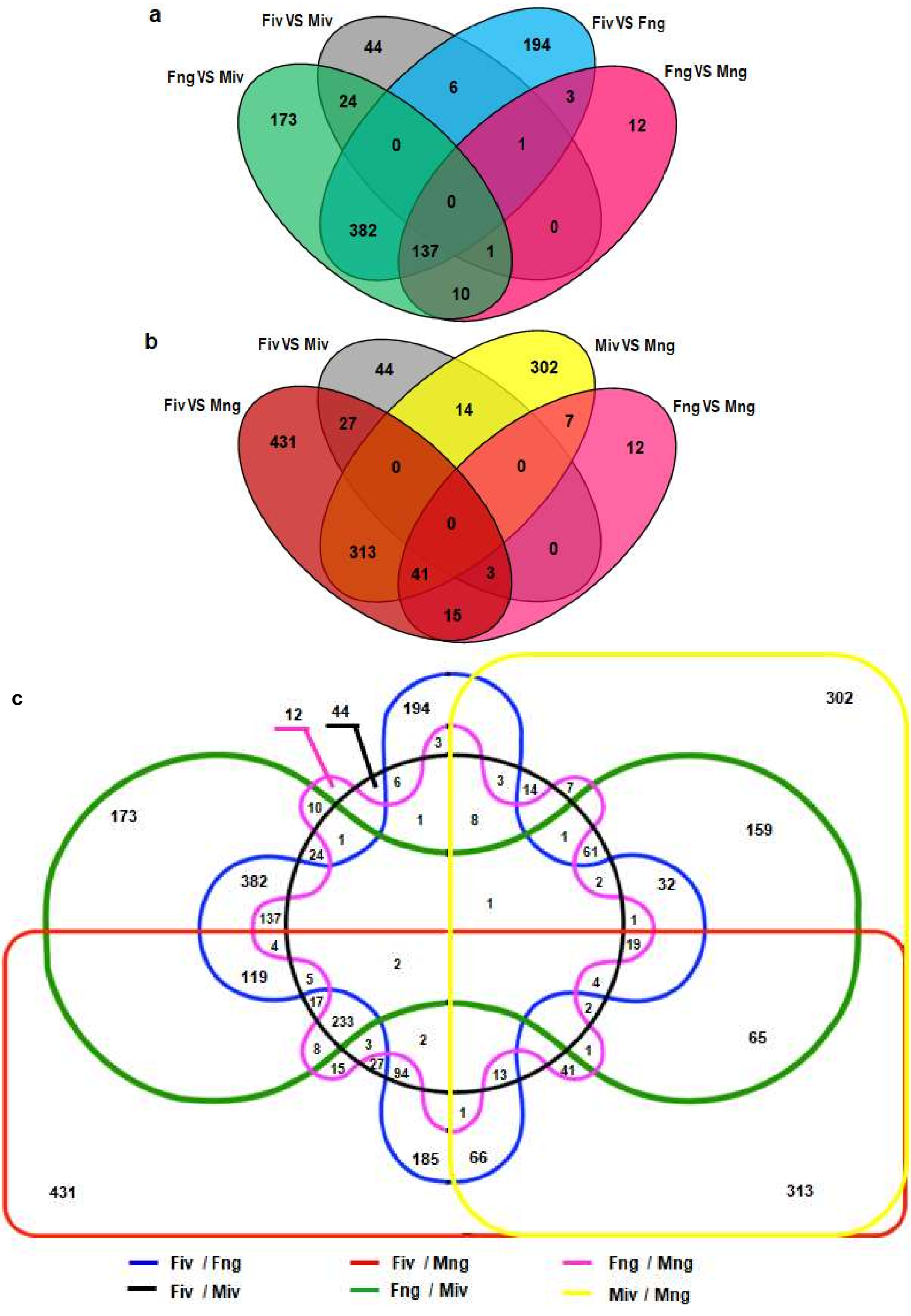

Fig. S5 Venn diagram showing co-expressed and uniquely expressed genes for *P. endiviifolia* different thalli types in the pairwise comparisons: (**a**) four pairwise comparison of female-specifically expressed genes connected with growth conditions and archegonia presence, (**b**) four pairwise comparison of male-specifically expressed genes connected with growth conditions and antheridia presence, (**c**) six pairwise comparison. Fiv – female thalli grown *in vitro*, Fng – female thalli with archegonia collected from natural habitat, Miv – male thalli grown *in vitro*, Mng – male thalli with antheridia collected from natural habitat.

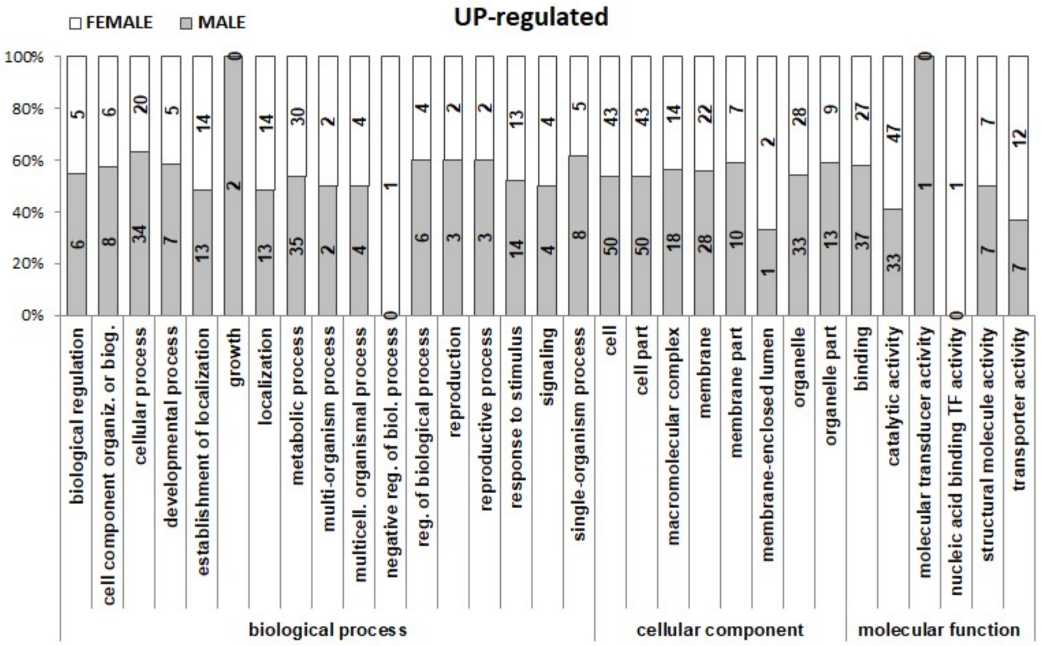

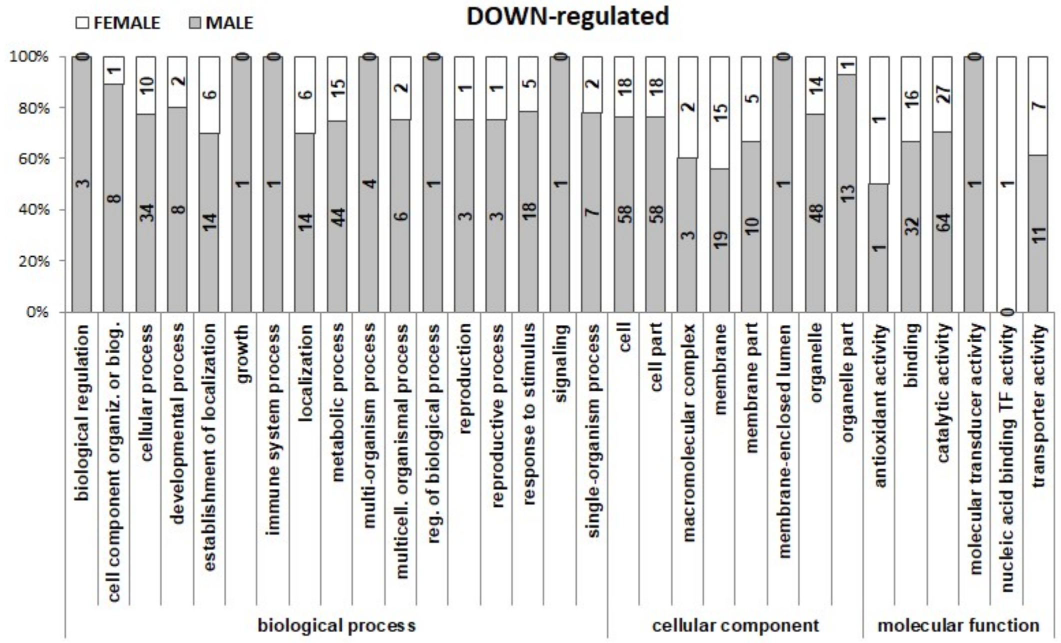

Fig. S6 Molecular function Gene Ontology classification of the genes identified as specifically changed in the sex-organ producing *P. endiviifolia* individuals. The results were summarized in the three main GO categories: biological process, cellular component and molecular function. The x-axis represented the functional category of the genes and the y-axis the gene numbers.

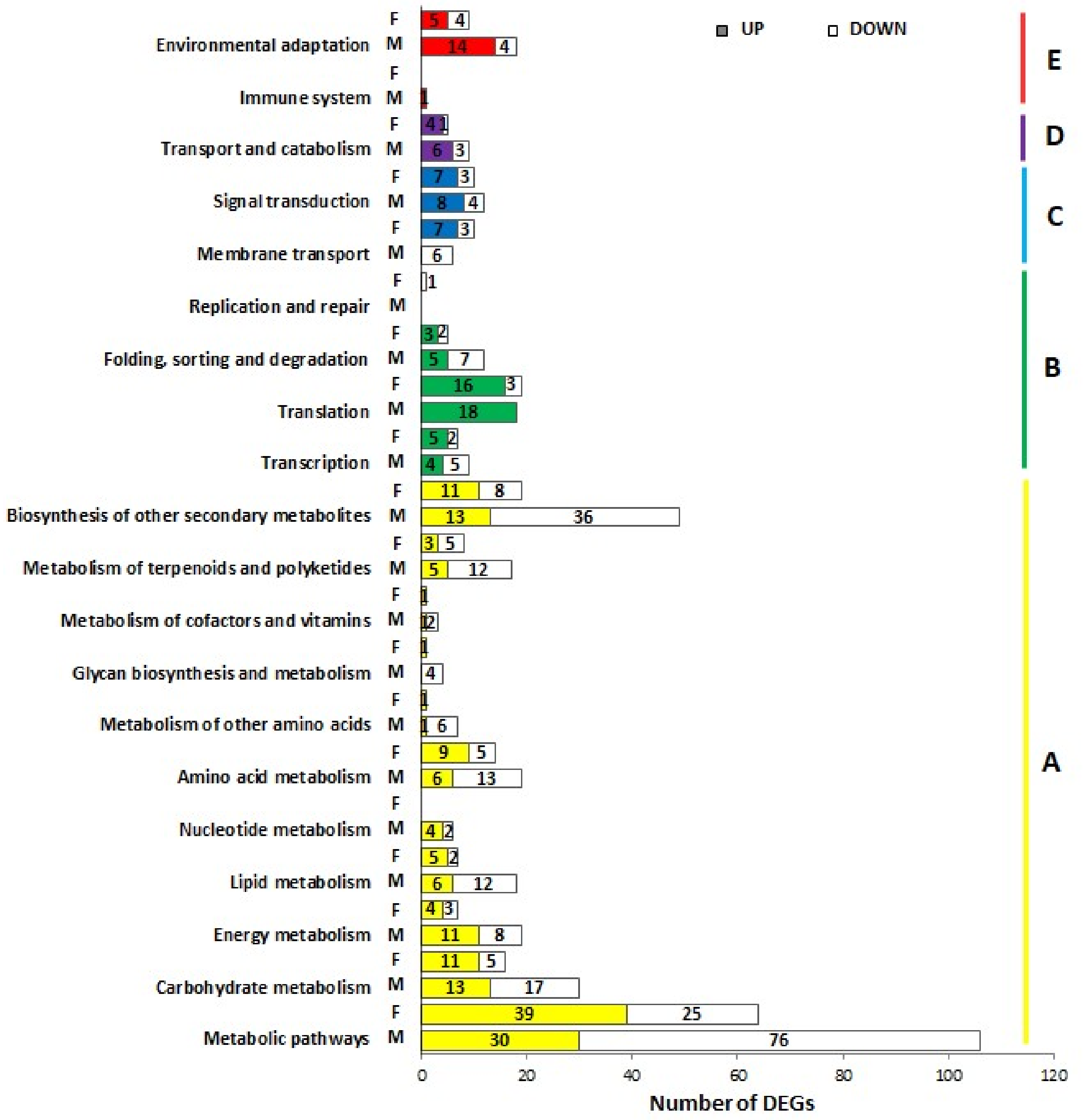

Fig. S7 KEGG enrichment analysis classification of the genes identified as specifically changed in the sex-organ producing *P. endiviifolia* individuals. (A) Metabolism; (B) Genetic Information Processing; (C) Environmental Information Processing; (D) Cellular Processes; (E) Organismal Systems.

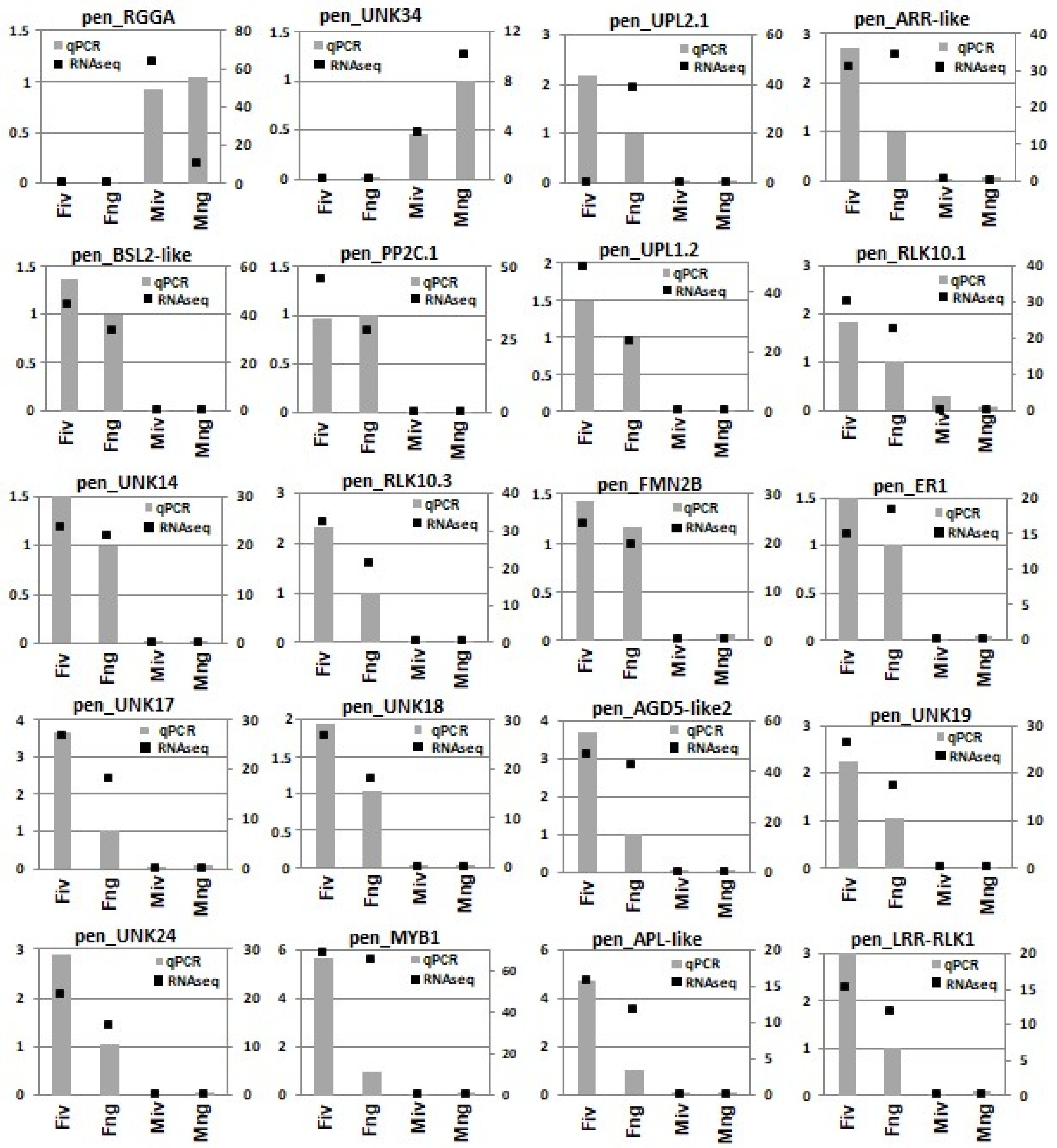

Fig. S8 Validation of additional examples of DEGs obtained by RNA-seq using RT-qPCR technique. Expression levels of selected genes in four types of *Pellia* thalli is shown: Fiv – female thalli grown *in vitro*, Fng – female thalli with archegonia collected from natural habitat, Miv – male thalli grown *in vitro*, Mng – male thalli with antheridia collected from natural habitat. Y-axis on the left side of graphs shows scale for RT-qPCR values (grey boxes); the relative expression of each gene was compared with female gametophytes grown in natural habitat and possessing archegonia, normalized against *ACTIN1* gene from two biological replicates. Y-axis on the right side of graphs shows scale for RPKM values (black squares) from NGS.

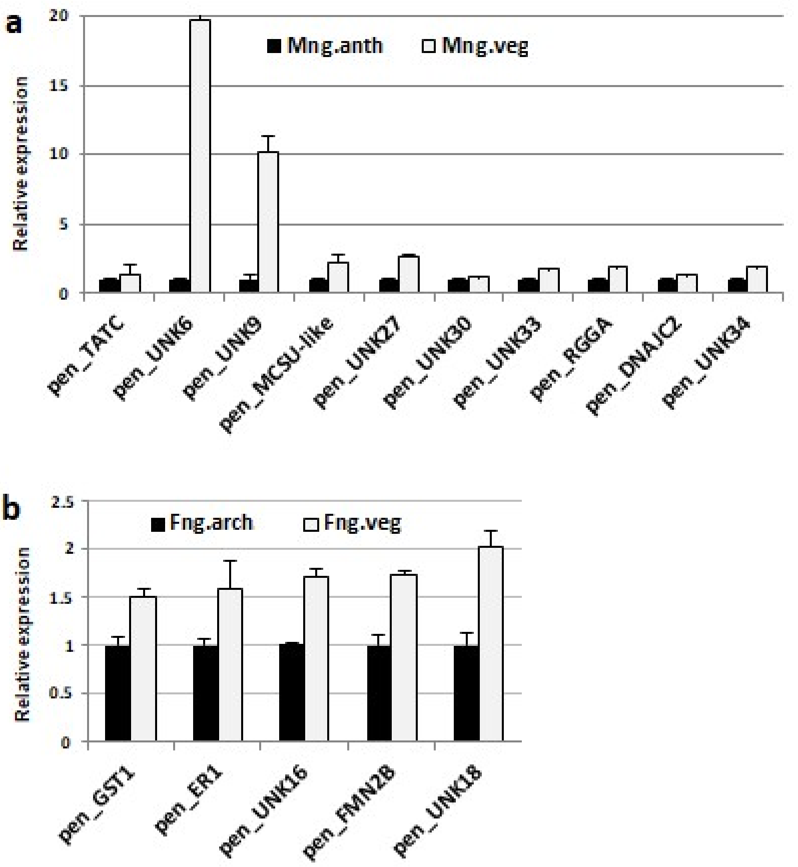

Fig. S9 Validation of the RNA-seq selected DEGs specific expression in *Pellia* antheridia or archegonia**.** Quantitative RT-qPCR analysis of the male-specifically expressed DEGs (**a**) and the female-specifically expressed DEGs (**b**) show different transcript levels in the vegetative and sex-organs bearing parts of the male and female gametophytes grown in natural habitat, respectively. (**a**) The relative expression of each gene was compared with antheridia (Mng.anth) isolated from the vegetative parts of male thalli (Mng.veg) grown in natural habitat. (**b**) The relative expression of each gene was compared with archegonia bearing region (Fng.arch) isolated from the vegetative parts of female thalli (Fng.veg) grown in natural habitat. All transcript levels were normalized against *ACTIN1*. Error bars indicate mean ± SD; n = 2.

References:

Morris JL, Puttick MN, Clark JW, Edwards D, Kenrick P, Pressel S, et al. (2018) The timescale of early land plant evolution. Proc Natl Acad Sci 115(10):E2274-E2283. https://doi.org/10.1073/pnas.1719588115

Pandey SN, Misra SP, Trivedi PS (2016) A Textbook of Botany Volume II, 13th Edition. Vikas Publishing Housen, Noida, India.

Paton JA (1999) The Liverwort Flora of the British Isles. Harley Books, pp. 525–530.

Schuster RM (1992) The Hepaticae and Anthocerotae of North America. Vol V. Field Museum of Natural History, Chicago.
