# Supplementary material for: The identification of differentially expressed genes in male and female gametophytes of simple thalloid liverwort Pellia endiviifolia sp. B using an RNA-seq approach
Source: Planta. 2020 Jul 15;252(2):21. doi: 10.1007/s00425-020-03424-z (PMC7363739; doi:10.1007/s00425-020-03424-z)
Supplement: Supplementary file 2 — Supplementary file2 (PDF 1154 kb) [file 425_2020_3424_MOESM2_ESM.pdf]

Supporting Data S2: Tables S1 – S6

The identification of differentially expressed genes in male and female gametophytes of simple thalloid liverwort *Pellia endiviifolia* sp. B using an RNA-seq approach

Planta

Izabela Sierocka[1]*, Sylwia Alaba[2], Artur Jarmolowski[1], Wojciech M Karlowski[2], Zofia Szweykowska-Kulinska[1]

[1] Department of Gene Expression, [2] Department of Computational Biology, Institute of Molecular Biology and Biotechnology, Faculty of Biology, Adam Mickiewicz University, Poznan, Uniwersytetu Poznanskiego 6, 61-614 Poznan, Poland

*corresponding author: Izabela Sierocka, email: izapaste@amu.edu.pl

Table S1 Summary of the clean reads mapping from the RNA sequencing results for four *Pellia endiviifolia* thalli types to the *Pellia* transcriptome; Fiv – female thalli grown *in vitro*, Fng – female thalli with archegonia collected from natural habitat, Miv – male thalli grown *in vitro*, Mng – male thalli with antheridia collected from natural habitat.

Sample ID	Total reads	Total mapped reads	Perfect match	≤ 2 bp mismatch	Total unmapped reads
**Pellia_Fiv**	42,198,064 (100%)	39,223,107 (92,95%)	33,924,111 (80.39%)	5,298,996 (12.56%)	2,974,957 (7.05%)
**Pellia_Fng**	45,740,368 (100%)	41,513,957 (90.76%)	35,581,346 (77.79%)	5,932,611 (12.97%)	4,226,411 (9.24%)
**Pellia_Miv**	42,117,686 (100%)	39,287,871 (93.28%)	33,606,044 (79.79%)	5,681,827 (13.49%)	2,829,815 (6.72%)
**Pellia_Mng**	45,909,691 (100%)	40,682,551 (88.61%)	34,984,031 (76.20%)	5,698,520 (12.41%)	5,227,140 (11.39%)

Table S2 Oligonucleotides used in the experiments.

**Oligonucleotides used in RT-qPCR experiments.**			
**Rank**	**Unigene ID**		
1	***Unigene12127**	ATTTTTGTCGGGCACAGAAG	U12127_F
		CCGTTCTTCAACAGGACGAT	U12127_R
2	**CL993.Contig14**	GACCGTGTTTTGGCTACGAT	CL993_F
		CCACCAAACACACAAAGGAA	CL993_R
3	**Unigene3827**	GCCCAGCCAAGTTATACCAA	U3827_F
		GTCGGCGTTGATTCTCAAAT	U3827_R
4	***Unigene6417**	TGCGTTCCATATCCAGATGA	U6417_F
		ACTGCGACAAATCGTGGAAT	U6417_R
		GGAGGACTCTGATCGAAGCA	U6417_2F
		TGGTAAGCAAATGCACCAAA	U6417_2R
5	***CL10631.Contig1**	GTCTCCTTCCGCTTCTGATG	CL10631.1_F
		GCCTGTGAAGACTGGTGTCC	CL10631.1_R
6	**Unigene3462**	TCGTCGCATCAGAATTGAAG	U3462_F
		CCAATTCGACACCCTGAAGT	U3462_R
7	**CL2052.Contig1**	AAAGAAGACATGGCACGCTTA	CL2052.1_F
		GAACCTTTGTGGGCCTGATA	CL2052.1_R
8	***Unigene9390**	CTGGCAGCAGTGTTTCGATC	U9390_F2
		CCGTCGCCTTTTATTGCTGA	U9390_R2
9	**CL3341.Contig1**	CCTTCCAAAACTGCCATTGT	CL3341.1_F
		CAGCTTCACATCTGCCTGAG	CL3341.1_R
11	***Unigene24732**	CGTTCTCTGCCTTCGGAACT	U24732_F2
		CATGCACATCTTCTCAGCGC	U24732_R2
12	**CL2052.Contig2**	GTTCTGCGACAAGGTCAACA	CL2052.2_F
		GGATGCAGATGAAGAGAAGCA	CL2052.2_R
13	**CL2625.Contig1**	TGCCAAAGAGGATTCTGGAG	CL2625.1_2F
		TTGGATACGGTTGCGTGTTA	CL2625.1_2R
14	**Unigene3025**	GTCTGGCCCAAAAGTAACCA	U3025_F

		AGCCTTGCAGCGTCCTATTA	U3025_R
**16**	***Unigene4874**	GCCACCAGCGTTTAAAATTC	U4874_F
		ACGTTCTCGCTTGTGGTCTT	U4874_R
		CTGAGCAGGAGAACCGAAAG	U3025_F2
		TAAACGCTGGTGGCTTTACC	U3025_R2
**17**	***Unigene34063**	TGCGAACGTAATTAATCAATGC	U34063_F
		TCGATTCAAACGAGAAAAGG	U34063_R
		TTCTTTTTCTTTTTGCGAACG	U34063_F2
		GTGGCTTAGATGGGACTGGA	U34063_R2
**19**	***Unigene29760**	GAGCCATTCATGAAACTTGGA	U29760_F
		CACTCAAAATCAACGCACGA	U29760_R
		TGTTTTTCCACCACCAGATG	U29760_F2
		CCAAGTTTCATGAATGGCTCA	U29760_R2
**20**	***Unigene4240**	CGTAATGCTAAAACGCATGG	U4240_F
		ATTCCCGGAAACACTGGAG	U4240_R
		GGTGAAAGATGTCCTCGTAATGC	U4240_F2
		GGAAACACTGGAGCTGGATATC	U4240_R2
		CCATGACGATAGTTTCAAATGG	U4240_3F
		TATCATCCATGCGTTTTAGCA	U4240_3R
**21**	**Unigene3842**	CCGGGAAGAGAAATGAGAGA	U3842_F
		GCTCTATATGCGGAGCTGGA	U3842_R
**22**	***CL7575.Contig3**	CCTCGAAAGTCTTCGCATTC	CL7575.3_F
		GTCCAACAGGGGTAAAGCAA	CL7575.3_R
**23**	**CL2052.Contig3**	CCCATGTTGCAGAGAAAGGT	CL2052.3_F
		TATTAGGCGGGGTGTTTTGA	CL2052.3_R
**24**	***Unigene27205**	TCCCGAATATGCTGAACCTC	U27205_F
		CGTCGTAATCTTCGTCGTCA	U27205_R
		ACTGGCCAAAATCCACCGAT	U27205_F2
		AGCCAGTGGATCGGAGTAGT	U27205_R2
		TCCGATCCACTGGCTAACTT	U27205_3F
		AATCGTTTTCAGCAATGCAA	U27205_3R
**25**	**Unigene38684**	ACGCTTTTTCATTCGACAGC	U38684_F
		CTTCCCGCAATTTACTTCCA	U38684_R
**26**	**Unigene43178**	CTCACCTTGGTTTTGGTGGT	U43178_F
		GACCCATCGGAAGAAGTCAA	U43178_R
**27**	**Unigene13624**	TCCGAAACCAAGTACGAACC	U13624_F
		AGGCAGTTGAGAGCGTGACT	U13624_R
**28**	***Unigene8335**	CTCCTTGAGGTTGGAAACCA	U8335_F
		CAGCGAATCAGGACAGTTGA	U8335_R
		GCAAATGGGCTCCTTGAGG	U8335_F2
		ACGCCTATGAAACCAGCCAG	U8335_R2
		GACGCCGTTTAACATCAGGT	U8335_3F
		TGGTTTCCAACCTCAAGGAG	U8335_3R
**29**	**CL270.Contig1**	CGCTAATCCTCTGGCAGAAC	CL270.1_F
		AATGGGAGCTTGGATCTGTG	CL270.1_R
**30**	**CL270.Contig2**	CTTCCTGGCAGAATGAGCTT	CL270.2_F
		CAGCCACTTCCTGGTTGAGT	Cl270.2_R
**31**	***CL9543.Contig2**	CAGTCTCCGAGGGTACGAAG	CL9543.2_F
		GCGAGGAGTCGAAGAACAAC	CL9543.2_R
**32**	**Unigene3709**	TGCTTGAGCACCTTCACATC	U3709_F
		TTGCCGCCTCTATGTAATCC	U3709_R
**33**	**Unigene37991**	TTCCGGTTCTACAAGGATGG	U37991_F
		ATGTGGGAATGGTGTCCCTA	U37991_R
**35**	**Unigene23723**	CACGTAGACTCGTTCGGTGA	U23723_F
		TTTTGGAGCAGCGAGTAGGT	U23723_R
**36**	**Unigene38620**	ATTTCCACCCAACAATCTGC	U38620_F
		TGGGAAAGCCTACCTCTTCA	U38620_R
**37**	**Unigene34156**	TTTTGAGCCATGATGAACGA	U34156_F
		TGGGGACATGGAAATTAACG	U34156_R
**38**	**Unigene9881**	TGGGGAGAAGTCCAAAGAGA	U9881_F
		CCGTTTGAGCTCTTTCATGG	U9881_R
**39**	**Unigene37231**	CGTCCTATTCCTGGTGATGC	U37231_F
		GAACAACTCAGCCGTGAATG	U37231_R
**40**	**Unigene31067**	ATGGGGTGAAGTCTGGCTCT	U31067_F
		GGTGCAGAAGGAGAAGCAAC	U31067_R
**41**	**Unigene40839**	CTCGTAATGCGAAGCATGTC	U40839_F
		AGCGGTTCCAAGCAACTCTA	U40839_R
**42**	***Unigene33541**	TGATGGACGTCTCCTCGACT	U33541_F
		CAAGCTCTCGTTAAAAATCCTTT	U33541_R
		AAACGAAGTCATAGCGTTTTCT	U33541_2F
		GTTAGTCGAGGAGACGTCCA	U33541_2R
**43**	**Unigene35474**	TAGGTGTTGGGGCAGTTCTC	U35474_F
		TTCGGTAATTGTTGCAAGGTC	U35474_R
**44**	***Unigene21321**	GACCCGGTAATGCCAATATG	U21321_F
		TGGAAGAACTGGATCGTCGT	U21321_R
		GTATGATCCAGCACCCGAAG	U21321_2F
		GCGGTTTGTTCGTGGTTAGT	U21321_2R
**45**	**Unigene5562**	AAGCTGGTGCAGAAAACCATA	U5562_F
		CTCAAAGTGGACAACCAGGAA	U5562_R
**46**	**Unigene23911**	GAAGAGGGAATGCTGATCCTC	U23911_F
		GACTACGGCAGCTACTGATGC	U23911_R
**47**	**Unigene41937**	CCTTCTGCACTCATCCCAGT	U41937_F
		AGTTTCCAGGAGCGAGTGAGT	U41937_R

48	Unigene9233	GTTATGCACTCCCAACTCCAA	U9233_F
		TGCTGATGATGTGAGACCTG	U9233_R
49	Unigene38557	TGATCTAAAGGCTCTGGCAAA	U38557_F
		GGCAACCAAGTCCTGTTACAA	U38557_R
50	Unigene42677	GCAGCACGTTTTGGAAAGTT	U42677_F
		TCCACTCGTATGCAGGGATAG	U42677_R
51	Unigene20525	AAGCAACTCCCAAGTCAGTCA	U20525_F
		AGTTGCTTTCCCAGTCGAAAT	U20525_R
52	Unigene40847	GCACAGTTATCCATTCCTCCA	U40847_F
		GATTTTTGGGGACAAGGTCAT	U40847_R
53	CL9235.Contig1	GCCTTCTCTCTTCGGTTCATT	CL9235.1_F
		ACCGCCAAAGCGTAAGAATAC	CL9235.1_R
55	Unigene11149	ACTCAGTTTGGACCCTCTGGT	U11149_F
		GGTCAGTTCCTACCCCTCTTG	U11149_R
56	*CL7475.Contig1	AGAGTCGTCAGGAAGCCCTAC	CL7475.1_F
		TGAGCTCGCTACCTTGTCATT	CL7475.1_R
57	Unigene24488	ACAACAATCACCTTCGCAATC	U24488_F
		CATGATGCAGTTGTTGAGCAT	U24488_R
59	Unigene5284	TTTGGAAAGTCAGCATCAGGA	U5284_F
		GGCTGTCTCTAGCATTTGTGG	U5284_R
60	Unigene11397	GCGTAGCTGGAGATGTCTTTG	U11397_F
		TCATTGTCTGATGGAGGAAGG	U11397_R
61	Unigene26727	TGCTTTGTACAGGCTTCACG	U26727_2F
		CCATGGGAAGAACCATTGAG	U26727_2F
62	Unigene4454	CATAAGGTAGCACCCCTGTCA	U4454_F
		TATCTGGGCACAAACCACTTC	U4454_R
64	Unigene10513	GCCACGTGCTTGATATTGATT	U10513_F
		TATCGCTACTTTCCCTGCTGA	U10513_R
65	Unigene23273	TCGGCAGACCTCTCAAAACTA	U23273_F
		CTCCCCAAGTCTAGCATTTCC	U23273_R
66	CL284.Contig1	CCTAACATGGCAGACCAACAT	CL284.1_F
		TTGCTTGCTCAATTTCTCGAT	CL284.1_R
68	Unigene19707	AAGTCACCTCCCGGTACAACT	U19707_F
		TTCCCAAGGATGTTAATGCTG	U19707_R
71	*Unigene37327	TCTTGAACTCCTGACCACCAG	U37327_F
		TCCAACAACAAGGTCAAAACC	U37327_R
		CCTTGTTGTTGGAGTTCTGACC	U37327_2F
		CTGCTGCTAAGAACGCCCA	U10597_2R
72	Unigene10597	TCTGCCACCGTGAACATAGTC	U10597_2F
		TGTTCTCACCTTCACCATGGAG	U10597_2R
10	Unigene19581	AGGGGTGAAGCCAAAGTTCT	U19581_F
		ATCCCAAGCCCCAAAGATAA	U19581_R
15	*CL691.Contig1	CCTCGACCATACCGAGAAAG	C691.1_F
		TCTTTGGGAGATTGCTGGAC	C691.1_R
18	*Unigene13259	TTTGGTTCCTGGTTCCTGTC	U13259_F
		AGCGACTACACGGTCGAGAG	U13259_R
34	Unigene30783	TCAGGATAGCGAGCTTGGAC	U30783_F
		CAGGCAGAGGAGCAAAGAAG	U30783_R
54	Unigene40405	CCCACATTTGTTAGCAGAACC	U40405_F
		GTGCGTTTCAACTGACAGGA	U40405_R
58	Unigene31174	TCCTTGTCCTGGAGTCTGCT	U31174_F
		TCCTCAACAGCAAGTGCAAC	U31174_R
63	CL10404.Contig1	ACCCAAGACGACTGCCATAG	C10404.1_F
		GTAGTAGAACCCCGCGATGA	C10404.1_R
67	CL11009.Contig1	CCACGATCTTTTGAGCGAGT	C11009.1_F
		TCCGGATGGAACTCTTATCG	C11009.1_R
69	CL3795.Contig1	ACGGGAAATGAGTCGAACAC	C3795.1_F
		TGGAAATTGCAGAGATGACG	C3795.1_R
70	Unigene14123	GATGATGGATGCCCCTTGTA	U14123_F
		AGCGGTACAGGTGAGATGTTC	U14123_R
**Oligonucleotides used in RACE experiments.**			
**Rank**	**Unigene ID**		
2	CL993.Contig14	GCAGCAAACGATAACCACCAAACACA	5_2
		GAGCCCGGATCATAGCTCACACAGAC	5N_2
		CTACGATTCTTTGATGCTGCGCTCGT	3_2
		GCGCAATGGGTTGTGGTCTATGTTTC	3N_2
3	Unigene3827	ATTTGAGAATCAACGCCGACCTTGTG	3N_3
		AGCTGCCCAGCCAAGTTATACCAAGC	3_3
		AACTTCCAGTTCACCATGGGCATCAC	5_3
		CTCACTGGAGTCTTGGATGGGCTCAC	5N_3
6	Unigene3462	GGTCGCTCATTACCAGGTTTCGGTCT	3_6
		TGCAACTCGTCGCATCAGAATTGAAG	3N_6
		GACCGAAACCTGGTAATGAGCGACCT	5_6
		ATAGGCGAATTGCATTGTGCCCTCTT	5N_6
8	*Unigene9390	ATCGAAACACTGCTGCCAGGTGCTAA	5N_8
		GGTTCCGTCGCCTTTTATTGCTGAAC	5_8
		GTAGCGAGCTTAGCACCTGGCAGCAG	3N_8
		GCCAGGTTAGGGCTGTTTATGAACTCG	3_8
9	CL3341.Contig1	ACCCAACTTTTCTCCCTCGGTCTGAAGA	3_9
		GTCTCAAACGTGCAACCTCGCCGCAT	5_9
		TCTTCAGACCGAGGGAGAAAAGTTGGGT	5N_9
11	*Unigene24732	GTCGAAGACATTCGGAGTGACCAAGC	5_11
		TTGTCGACGACCTCCTTGTTGAAGGT	5N_11

		GCTTGGTCACTCCGAATGTCTTCGAC	3_11
		CTCTGCGTTCTCTGCCTTCGGAACTA	3N_11
**13**	**CL2625.Contig1**	ATTGAACCACCAAAGTTCCCCTGCTG	5_13
		AGGCAATTGGATACGGTTGCGTGTTA	5N_13
		CAAAGCTGTGCCAAAGAGGATTCTGG	3_13
		TTTCACGGGAGAATGTGGCAAGTCAT	3N_13
**14**	**Unigene3025**	ACCAGCCCTCCTAATGCACCACCTGA	5_14
		TCCTGAAAGGGATGGTGGAGCTGAT	3_14
		AAAACGGCTTTTGGACAGGCAGATG	3N_14
**21**	**Unigene3842**	GCCATTTCATTTTGGGCTTGCAGTT	3N_21
		TGCGGAGCTGGATTTCAGAATACACA	3_21
		GCACTCATCAAATCAACCACCACGA	5N_21
		CCAGCTCCGCATATAGAGCTCTCAGCA	5_21
		TGGCCACCATCCCACTCTTGCTTGG	5N_14
**22**	***CL7575.Contig3**	CCCTTGACATGTTGTCGGTTCTTTGG	3_22
		GAGGCCATGGAAGCCGAGAAGTCTTA	3N_22
		CCCAAAGAACCGACAACATGTCAAGG	5N_22
		GGAGTTCCTCCACCTGGGAAATGAGA	5_22
**25**	**Unigene38684**	CTGTCCGGCCGGCTGTTCTATAGTCT	3_25
		TATCACACGGGTGACACAGGTCGATT	3N_25
		CCTCGTGCCACCAGAAAATCAATCAC	5N_25
		CGACCTGTGTCACCCGTGTGATAC	5_25
**26**	**Unigene43178**	TGCGACTGCTGGAAGGAGAAATTGA	3_26
		CCTCACCTTGGTTTTGGTGGTGGTT	3N_26
		CCGATGGATGATGTGAGGGATGAAA	5N_26
		GCTTGCCTTCATATCCCGATGGATG	5_26
**27**	**Unigene13624**	GCCTGGACAAACAATGCACAACTCAA	3_27
		AAGCTCTTCGACCTTCCCATGCATCT	3N_27
		GTCCAGTTGAGATGCATGGGAAGGTC	5_27
		GCACCAGCACAAGGATGTTGTCAAAG	5N_27
**30**	**CL270.Contig2**	TCAGATGGAGGGTGTCGAGGCAGGAAG	3_30
		CCTGGGAAACTGAAGGAGCCATTGAGCA	3N_30
		TTCCTGCCTCGACACCCTCCATCTGA	5N_30
		GCTCAATGGCTCCTTCAGTTTCCCAGGT	5_30
**32**	**Unigene3709**	TGGGGACTCTCGTGCTGTGCTTTCGC	3N_32
		GGGAAATGGAGGATGCCGTGACAGCT	3_32
		GTTTCGTGCTTTCCCTCCGGCGACAC	5_32
**33**	**Unigene37991**	AACCCTTCCGGTTCTACAAGGATGGA	3_33
		ACCAGGTAGGGACACCATTCCCACAT	3N_33
		CCTCTCCATCCTTGTAGAACCGGAAGG	5_33
		CGATGCTTATTGTGAATGGATAGGTTAG	5N_33
**35**	**Unigene23723**	TTGGAGTTGAAACACTGCCGTGAATG	3_35
		TTGGAGCAGCGAGTAGGTTGAAGCTG	3N_35
		AGCGACATACTCCCATGCACTGTCG	5.2_35
		CATTCACGGCAGTGTTTCAACTCCAA	5N_35
**36**	**Unigene38620**	TGAACGTGCGAAGGTAGTTGATGGTG	3_36
		ATCCTGTTGGCAGTGATGCAGAGGAC	3N_36
		CACCATCAACTACCTTCGCACGTTCA	5_36
		TCCTGTGGTCATCATCCGATCCTCTT	5N_36
**37**	**Unigene34156**	TGTCCCATTCCATACGCCCAGGATCCG	3_37
		GGGTCGGATCCTGGGCGTATGGAATGG	5_37
**38**	**Unigene9881**	GATTGAGCTTCTTGGCTCCCTTGGTG	5_38
		ATGGTCCCCTGCTTGACCTTCATTTC	5N_38
		GCCGGCAACAAGGAAAATACACTAGCA	3_38
		TCCAAAGAGAGAAGGAGCGGTACAAGC	3N_38
**39**	**Unigene37231**	AAGGAAAGGGTGGTCGACGTACCAAA	5_39
		ATGGGAGAAGAACAACTCAGCCGTGA	5N_39
		GCATCTTCGTCCTATTCCTGGTGATGC	3_39
		GTGGGTATGAAGTCCGCTTGTTGTGC	3N_39
**40**	**Unigene31067**	TCTTCTGGACCCATCTCCTTGCTTCA	5_40
		CACTCGGACGTTTGAAATTTGGAGGA	5N_40
		CCCCATCCACTCCTGATGAAAAGTCA	3_40
		CTCGCTGTGTCTGGTCAAAGAGCTGA	3N_40
**41**	**Unigene40839**	ATGGAGCTGTGGTTGTCGAGCAGTCT	5_41
		TCCAAGCAACTCTACAATTGGCAGCA	5N_41
		AAAGTTTGGTGCACGGCATGTAGCTC	3_41
		GATCAACATTCCCTTGCCCTGGAGTT	3N_41
**45**	**Unigene5562**	CAATCCTCTCCTTCAGTGGCACAAGG	5_45
		TCCTCAAAGTGGACAACCAGGAATGC	5N_45
		GGAAGCTGGTGCAGAAAACCATAGAAGG	3_45
		TGGAAGAATCACAATCTGGAGGCAGAA	3N_45
**47**	**Unigene41937**	TTACGGAGTTTCCAGGAGCGAGTGAG	5_47
		TTACGACCATAGGCCCTGCTTGTCTG	5N_47
		TTCTGCACTCATCCCAGTTACCAGCA	3_47
		CAGACAAGCAGGGCCTATGGTCGTAA	3N_47
**48**	**Unigene9233**	TGGGGAGGAGAAGGTCTGGGATAGGT	5_48
		GAAGTCCATTCACAAAGACCGGGACA	5N_48
		TGGTCCTGTTATGCACTCCCAACTCC	3_48
		TCAGCACCTCAACAGCACCATTTGTC	3N_48
**49**	**Unigene38557**	CTAAAGGCTCTGGCAAAGGAGGAGGA	3_49
		CATTCATAATGGTTGGACCCACAAATCC	3N_49
		CGGATTTGTGGGTCCAACCATTATGA	5N_49
		GGCAACCAAGTCCTGTTACAAATGTGC	5_49

**50**	**Unigene42677**	GGAGAGAGAGACGCCGCCCTTCTTCG	3N_50
		TGACAATGCACAACTTGACTCTCGGCGG	3_50
		ACGATCACAGTCGAGTCTCCGCCGA	5_50
		CATCCTGTGAAACGGGTGCTTTAAG	5N_50
**51**	**Unigene20525**	GAGAAGGCCCGAGAAACTCTTGCTGGG	3_51
		TTCAGGAGCTCTCGGATTTGGCCTCC	3N_51
		TGGTCCTGGTTCTTGTGCGGGCAGAC	5N_51
		CGGCGTCCACAAACCGTTCGTGTAGTT	5_51
**52**	**Unigene40847**	GTCATCTTTGCGAGTGGAGGAATGGA	5_52
		CTGTGCTTTCTTGGCATTCCTTGTCG	5N_52
		GTAAAGCCGTGAATCACATGGGCAAA	3_52
		CCTTCGCTCTAAGGAAGTCCCCATGT	3N_52
**55**	**Unigene11149**	AAGCTCTCTCCCGGTAGAAGCACCCA	3_55
		ACGGATGTGCGGTTGTCATGCTCCT	5N_55
		ACTCCCACCAGACCCTGCCTTATCATCA	5_55
**56**	***CL7475.Contig1**	CAGTCTCATTCAGCCAGTTCCGTGTG	5_56
		GAAGTCAAGGCCACGGCAACAAGTTA	5N_56
		AACGCATAATCCTTGAGCCACAGCAC	3_56
		TAACTTGTTGCCGTGGCCTTGACTTC	3N_56
**57**	**Unigene24488**	TTGCGGTTGAGAAGATGGACTGGACT	5_57
		TGCTGCTGGGGGTGAGAGTTAACAAG	5N_57
		CTCTATCACAGGCACCTCAGCCCTTG	3_57
		CACCATCCGCAAGGTCATCTTAATGC	3N_57
**59**	**Unigene5284**	CCAAAGGAGACGGAGGCAATTTCAAG	5_59
		TTACATGCATGGCTCGCAGAGGAGAT	5N_59
		CCCATGGTCACACACCATGTATCCAC	3_59
		AGGATGTTGAGAGGCTCTGCATGGAA	3N_59
**60**	**Unigene11397**	CCAGTGCAGGCTTCAACCACGGTACC	3_60
		CGTGTGTGCATGTCCAAAGTCGCCCA	5N_60
		AGCTCTGTCTGCACCACCAAGCTCGC	5_60
**62**	**Unigene4454**	ACCCAGCTGTATCTGGGCACAAACC	3N_62
		CAGCTTGGGAAGCTCTTCAACCTTCAG	3_62
		TGGAAGTGGTTTGTGCCCAGATACA	5N_62
		GGTAGCACCCCTGTCAAATGGCTTG	5_62
**64**	**Unigene10513**	TGTTGGCCTCGCAGCAGACACAGACT	3_64
		GAGCTGCAAGTCATCCTCCGCCACCT	3N_64
		AGTGCCTTCAAAGCAGTGGTCCCAACA	5_64
		TCCTACCACACCCCTGTCCATTGCCG	5N_64
**66**	**CL284.Contig1**	CTTGGCCTACACAATCGGGTGCTAGA	3_66
		GCAGACCAACATGGGAACGTCAGATT	3N_66
		TCTAGCACCCGATTGTGTAGGCCAAG	5_66
		GGCAAGGGTGGGTCCATCACTACATT	5N_66
**68**	**Unigene19707**	GTATCGTGGTGATGTGGTTCCCAAGG	3_68
		CTGCTGTGGCCACCATTAAAACCAAG	3N_68
		CTCCCGGTACAACTGTAGGTGGCTGA	5N_68
		GCACGCTTCGCATACATGAGATCAAA	5_68
**10**	**Unigene19581**	TTTATCTTTGGGGCTTGGGATGAACC	3_10
		TCGTGGATCAGACGTGGTAATTGTGA	3N_10
		GGCTTGGATTCGGGGACCATAACTTT	5N_10
		TGCGAACACTCACAATTACCACGTCTG	5_10
**15**	***CL691.Contig1**	GCAGGTGCTGCCATAAGAGACAGTCC	3_15
		CAGCCCTCGACCATACCGAGAAAGA	3N_15
		CCGGGTTCTTTCTTTGGGAGATTGC	5_15
		CTTGATGCAATGCTCCCTGAAGTGG	5N_15
**18**	***Unigene13259**	GTTCCTGGTTCCTGTCGACCGTATAG	3_18
		TAGTCCGTTATGAGTTTCTGGTTCCTG	3N_18
		CGGTCGAGAGGAATAGGAACCAAGAAC	5_18
		CCAAGAACAAACTCAGAAGCGACTACAC	5N_18
**34**	**Unigene30783**	GGCAGAGGAGCAAAGAAGAGGAACCA	3_34
		CCGATCACCTCGACAGAAATGCGATA	3N_34
		GTGGCACAGGATTTGACCCCATCTT	5N_34
		GCCTTTGGTTCCTCTTCTTTGCTCCTC	5_34
**54**	**Unigene40405**	TGGAGCAACCCACATTTGTTAGCAG	3_54
		TGAGAAACTAGGCTGCATTGCACACA	3N_54
		TCCCCAGATGACTCTCTCAGGTTCTCA	5_54
		AAGTGCGTTTCAACTGACAGGAAATGAC	5N_54
**58**	**Unigene31174**	CCAGGATGAGGCAGAGCGACAGAAGC	3N_58
		GTGTGACTGGTGAGCAGGCAGGAGGT	3_58
		GCTTGGAACCGCTTCTGTCGCTCTGC	5N_58
		AGCTTCCCCAGGTGTTAATGGGACAG	5_58
**63**	**CL10404.Contig1**	AGTCGTCTTGGGTTCGGAGAGAGAGG	3_63
		TGTCCCTCACCGGGAATCACAGTTAC	3N_63
		TCGATCGACGAGAGAGCTCGAGTAGC	5N_63
		TCTGCTAATAAGCCCCACCAGTGCAG	5_63
**67**	**CL11009.Contig1**	ATCGTGGTGCCTATGTCCGCTATCAA	5_67
		AAGCGTTTCTCCAAATGGGTGGAAGA	5N_67
**69**	**CL3795.Contig1**	GTCCCTATTCCACCACCGGAAACAAC	5_69
		AAGTGTGAGCCTCGGAAGTCCTCGAT	5N_69
**70**	**Unigene14123**	GTTTCAGCTCAAAATGCCATCGAGGT	5_70
		GCGGTACAGGTGAGATGTTCAACAGG	5N_70

Table S3 Expression pattern of RDA-cDNA identified male- or female-specifically expressed genes within the RNA-seq data; Fiv – female thalli grown *in vitro*, Fng – female thalli with archegonia collected from natural habitat, Miv – male thalli grown *in vitro*, Mng – male thalli with antheridia collected from natural habitat.

GeneID	Gene (Ac.No.)	Fiv RPKM	Miv RPKM	log2 Ratio (Fiv / Miv)	logFC.edgeR (Fiv / Miv)	Regulation	P-value edgeR	FDR edgeR
Unigene15626	*PenB_TUA1 HQ634388*	0,932632	420,58	-8,816857033	-9,236876	Down	7,37E-15	2,64E-11
Unigene16140	*PenB_Rab_a1/11* HQ634389	0,1251	68,3873	-9,09495608	-9,4842270	Down	3,09E-14	6,12E-11
**GeneID**	**Gene (Ac,No,)**	**Fng RPKM**	**Mng RPKM**	**log2 Ratio (Fng / Mng)**	**logFC,edgeR (Fng / Mng)**	**Regulation**	**P-value edgeR**	**FDR edgeR**
Unigene15626	*PenB_TUA1* HQ634388	1,668907	384,4757	-7,847845	-7,435291	Down	1,46E-11	9,48E-09
Unigene16140	*PenB_Rab_a1/11* HQ634389	0,269001	107,2837	-8,639601	-8,217021	Down	1,5E-12	1,64E-09
Unigene38805	PenB_CYSP KF853593	18,44132	0,088606	7,7013156	8,072883	Up	1,63E-11	1,00E-08
Unigene7398	PenB_MT2 KF853596	1,592183	0,01	7,3148624	9,257657	Up	1,41E-06	2,35E-04

Table S4 Number of DEGs specifically changed in the *P, endiviifolia* individuals producing sex organs; Fiv – female thalli grown *in vitro*, Fng – female thalli with archegonia collected from natural habitat, Miv – male thalli grown *in vitro*, Mng – male thalli with antheridia collected from natural habitat,

**MALE thalli producing ANTHERIDA**				**FEMALE thalli producing ARCHEGONIA**		
**DEGs list of two thalli comparison**	**No, of DEGs UP regulated**	**No, of DEGs DOWN regulated**		**DEGs list of two thalli comparison**	**No, of DEGs UP regulated**	**No, of DEGs DOWN regulated**
Fng vs **Mng**	3	9		Mng vs **Fng**	9	3
Miv vs **Mng**	100	202		Miv vs **Fng**	97	76
Fiv vs **Mng**	254	177		Fiv vs **Fng**	151	43
Common for:<br>Fiv vs **Mng**<br>Miv vs **Mng**<br>Fng vs **Mng**	30	11		Common for:<br>Fiv vs **Fng**<br>Miv vs **Fng**<br>Mng vs **Fng**	134	3
	All: 387	All: 399			All: 391	All: 125
	**Total DEGs No,: 786**				**Total DEGs No,: 516**	

Table S5 Summary of RACE experiments for transcripts with male- or female specific expression selected in RNA-seq experiment,

RANK No,	Gene ID	Length from RNAseq [nt]	Length from 5'&3'RACE [nt]	**annotation - blastx Marchantia polymorpha genome** (Protein sequences of the "primary" transcripts (version 3,1, November, 2017))
**2**	pen_UNK2	257	**523, 634**	-
**3, 9[a]**	pen_RRM-I	735, 236	**2071, 2006, 2488[INT]**	>Mapoly0011s0129,1 [KOG0131] Splicing factor 3b, subunit 4; [PF14259] RNA recognition motif (a,k,a, RRM, RBD, or RNP domain)
**6**	pen_GST1	783	**2697**	>Mapoly0019s0024,1 [GO:0005515] protein binding; [PF13417] Glutathione S-transferase, N-terminal domain; [KOG0406] Glutathione S-transferase
**7, 23, 46, 64[a]**	pen_UPL2	214, 364, 541, 869	**3049 [3R]**	>Mapoly0090s0059,1 [K10592] E3 ubiquitin-protein ligase HUWE1 [GO:0004842] ubiquitin-protein ligase activity;
**10**	pen_TATC	622	**854[5R]**	>Mapoly0010s0061,1 [PF00902] Sec-independent protein translocase protein (TatC);
**5, 8, 11[a]**	pen_CHc1	310, 202, 626	**1642**	>Mapoly0040s0034,1 [KOG4742] Predicted chitinase; [GO:0006032] chitin catabolic process;
**13**	pen_ARR-like	202	**2313, 2377, 2455, 2623**	>Mapoly0101s0006,1 [PF00072] Response regulator receiver domain; [PF00249] Myb-like DNA-binding domain;
**14**	pen_BSL2-like	1941	**3512**	>Mapoly0018s0002,1 [PF13418] Galactose oxidase, central domain; [PTHR11668] SERINE/THREONINE PROTEIN PHOSPHATASE;
**15**	pen_UNK6	466	**475**	-
**18**	pen_UNK9	271	**838, 1003**	-
**21, 32[a]**	pen_PP2C,1	940, 1933	**2851**	>MapolyY_A0044,1 [K14497] protein phosphatase 2C [EC:3,1,3,16]; [KOG0698] Serine/threonine protein phosphatase
**22**	pen_UNK12	710	**718**	-
**27**	pen_UNK14	999	999	-
**34**	pen_MCSU-like	635	**1722**	>Mapoly0093s0023,1 [PTHR14237] MOLYBDOPTERIN COFACTOR SULFURASE (MOSC); [KOG2142] Molybdenum cofactor sulfurase;
**35**	pen_FMN2B	366	**1644**	>Mapoly0138s0020,1 [PTHR23213] FORMIN-RELATED; [PF02181] Formin Homology 2 Domain;
**36**	pen_YIPPEE-like	611	**777, 921**	>Mapoly0011s0154,1 [PF03226] Yippee zinc-binding/DNA-binding /Mis18, centromere assembly
**37, 43[a]**	pen_ER1	452, 1103	**988, 1171**	>Mapoly0112s0015,1 [PTHR12373] ENHANCER OF RUDIMENTARY ERH; [PF01133] Enhancer of rudimentary
**38**	pen_UNK17	266	**280 [5R]**	-
**39**	pen_UNK18	217	**1444, 1820**	>Mapoly0028s0105,1 [GO:0016758] transferase activity, transferring hexosyl groups; [PF05637] galactosyl transferase GMA12/MNN10 family
**40, 25[a]**	pen_AGD5-like1	1235	**2392**	>Mapoly0041s0037,1 [GO:0008060] ARF GTPase activator activity; [KOG0702] Predicted GTPase-activating protein
**41**	pen_UNK19	884	**956**	>Mapoly0125s0028,1 [KOG0144] RNA-binding protein CUGBP1/BRUNO (RRM superfamily); [PTHR23189] RNA RECOGNITION MOTIF-CONTAINING
**45**	pen_UNK22	246	**483**	-

**49**	pen_TAO1-like	337	**5019**	>Mapoly0001s0394,1 [PF13855] Leucine rich repeat
**50**	pen_BPC6-like	929	**1681** [3R]	>Mapoly0018s0011,1 [PTHR31421] FAMILY NOT NAMED; [PF06217] GAGA binding protein-like Family
**51**	pen_MYB1	1238	**1836**	>Mapoly0098s0044,1 [PF00249] Myb-like DNA-binding domain; [GO:0003682] chromatin binding
**52**	pen_UNK25	329	**443**	-
**54**	pen_UNK27	250	**2468**	-
**55**	pen_SPL1	1838	**4700**	Mapoly0014s0224,1 [GO:0003677] DNA binding; [PF03110] SBP domain
**56**	pen_UNK28	1032	**1027**	-
**57**	pen_UNK29	360	360	-
**58**	pen_UNK30	629	**703, 1140**	-
**59**	pen_UNK31	431	431[5R]	-
**60**	pen_APL-like	1826	1826	>Mapoly0115s0048,1 [PF00249] Myb-like DNA-binding domain; [GO:0003682] chromatin binding
**26[INT], 29, 30, 62, 65, 72[a]**	pen_LRR-RLK1	1321, 238, 366, 419, 506, 308	**5819[INT], 6117**	>Mapoly0011s0213,1 [PF13855] Leucine rich repeat; [KOG1187] Serine/threonine protein kinase
**63**	pen_UNK33	321	**430, 456, 490, 849, 872**	-
**66**	pen_CUL3A	521	**2766**	>Mapoly0011s0201,1 [GO:0031625] ubiquitin protein ligase binding; [GO:0031461] cullin-RING ubiquitin ligase complex
**67**	pen_RGGA	2256	2256	>Mapoly0044s0032,1 [PTHR12299] HYALURONIC ACID-BINDING PROTEIN 4; [PF09598] Stm1; [KOG2945] Predicted RNA-binding protein;
**68**	pen_TUA2	374	**1595, 1871, 2025**	>Mapoly0066s0086,1 [PF00091] Tubulin/FtsZ family, GTPase domain; [KOG1376] Alpha tubulin
**69**	pen_DNAJC2	3349	3349	>Mapoly0114s0007,1 [KOG0724] Zuotin and related molecular chaperones (DnaJ superfamily),contains DNA-binding domains;
**70**	pen_UNK34	324	**379[5R]**	**-**

[a] RACE experiments revealed that those ranks originate from one mRNA; [5R] or [3R] 5' or 3'RACE products, respectively; [INT] intron containing transcripts**,**
ranks with male-specific expression; xxx mRNA isoforms with different polyadenylation sites,

Table S6 Expression profiles of *M, polymorpha* ortholog genes of *P, endiviifolia* genes specifically expressed in the antheridia and archegonia,

Rank ID	Pellia tracking_id	annotation - blastx nr NCBI	Annotation - blastx MarpolBase	RNAseq data (FPKM) [a]												Annotation by JGI
				antheridiophore	archegoniophore	sporophyte	sporeling (HATC)	sporelings 0hrs	sporelings 24hrs	sporelings 48hrs	sporelings 72 hrs	sporelings 96hrs	thallus, cut 24h	thallus, cut 0h	thallus 30C	
**5, 8, 11**	Pen_Unigene24732	gi|168059395|ref|XP_001781688,1| predicted protein [Physcomitrella patens] gi|124358661|dbj|BAF46006,1| putative class I chitinase, partial [Cryptomeria japonica]	Mapoly0040s0034,1	0,01237	65,4722	0,0534	0,0635	0	0	0	0	0	0	0	0,0426	[KOG4742] Predicted chitinase; [GO:0016998] cell wall macromolecule catabolic process; [PF00182] Chitinase class I
**55**	Pen_Unigene11149	gi|168046334|ref|XP_001775629,1| predicted protein [Physcomitrella patens subsp patens] gi|94448662|emb|CAI91303,1| squamosa promoter binding protein 1 [Physcomitrella patens]	Mapoly0014s0224,1	48,8775	88,4929	44,9418	1,9285	0,1255	0,0619	0,0168	0	0	23,7519	10,2738	7,8428	[GO:0003677] DNA binding; [GO:0005634] nucleus; [PF03110] SBP domain
	PenB_HMGbox	*PenB_HMGbox* HQ634391	Mapoly0031s0059,1	1577,31	0,1003	0,3469	1,611	0,0319	0	8,978E-07	0,0373	0	2,6995	0,2539	0,6840	[KOG0381] HMG box-containing protein; [PF00505] HMG (high mobility group) box;
			Mapoly0031s0059,2	7,4629	0	5,62E-06	0,1608	0,0728	0,0248	0,0325	0	0	0,4333	0,1136	0	[PF00505] HMG (high mobility group) box;

[a] FPKM values of *M, polymorpha* genes from the data published by Bowman et al, 2017,
